# Benzoxazine-based innovations in shielding: enhancing durability and protection in electronics and clothing

**DOI:** 10.1080/15685551.2026.2664957

**Published:** 2026-05-05

**Authors:** Ayisha A. M. Arishi, Khalid A. Alamry, Mahmoud A. Hussein

**Affiliations:** a Chemistry Department, Faculty of Science, King Abdulaziz University, Jeddah, Saudi Arabia; b Department of Physical Sciences, Chemistry Division, College of Science, Jazan University, Jazan, Saudi Arabia; c Chemistry Department, Faculty of Science, Assiut University, Assiut, Egypt

**Keywords:** Benzoxazines, wearable technology, electronic shielding military applications

## Abstract

Benzoxazine-based materials have attracted increasing attention for electromagnetic interference (EMI) shielding due to their excellent thermal stability, mechanical strength, low moisture absorption, and dimensional stability. This review presents a comprehensive overview of recent advances in benzoxazine-based nanocomposites for EMI shielding applications, with particular emphasis on the role of conductive and functional nanofillers, including multi-walled carbon nanotubes (MWCNTs), carbon fibers, Ti₃C₂ MXenes, nanoclays, and epoxy-based hybrid systems. The incorporation of these nanofillers significantly enhances electrical conductivity, thermal resistance, and mechanical robustness, enabling the development of lightweight and durable shielding materials suitable for electronics, wearable devices, and military applications. In addition, this review discusses key challenges related to processing, scalability, and environmental sustainability, as well as emerging strategies such as bio-based benzoxazine resins and low-temperature curing approaches. Overall, this review highlights the adaptability and long-term durability of benzoxazine-based systems and underscores their growing relevance as advanced shielding materials across diverse technological fields.

## Introduction

Electronics form the backbone of modern technologies, enabling communication, healthcare, industrial automation, and advanced defense systems. The rapid miniaturization and increasing operating frequencies of electronic devices have intensified concerns related to electromagnetic interference (EMI), which can adversely affect device performance, reliability, and safety [1–3]. Consequently, effective EMI shielding materials are essential to ensure the stable operation of electronic systems in diverse environments [4]. Traditional EMI shielding materials, such as metals, offer high electrical conductivity but suffer from inherent limitations including high density, corrosion susceptibility, and poor flexibility, restricting their applicability in lightweight, flexible, and wearable systems [5,6]. As a result, polymer-based materials have emerged as attractive alternatives due to their low density, ease of processing, and tunable properties [7].

Among polymers, benzoxazines have gained increasing attention as high-performance thermosetting resins owing to their excellent thermal stability, near-zero curing shrinkage, low moisture absorption, and superior mechanical properties compared with conventional thermosets [8–10]. These characteristics make benzoxazines promising candidates for shielding applications in electronics and protective clothing, where durability, thermal resistance, and long-term stability are critical.

**Figure 1.**
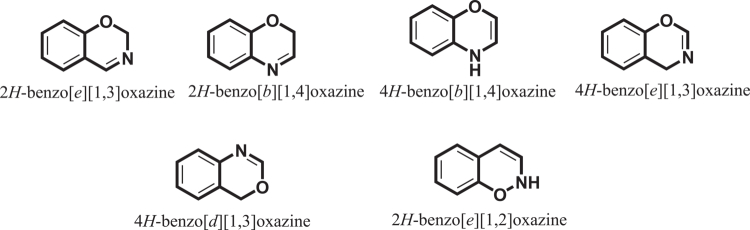
Chemical structure of a benzoxazine monomer. This ﬁgure has been adapted/reproduced from Ref. [11] with permission from Elsevier.

The resulting benzoxazine monomers subsequently undergo ring-opening polymerization upon curing to form highly cross-linked polybenzoxazine (PBz) networks [11]. To illustrate this fundamental structure, Figure 1 presents a general structural overview of benzoxazine monomers, serving as a basis for subsequent discussions on polymerization and composite design.

The unique chemical structure of benzoxazines enables structural versatility and property tunability, allowing the design of materials suitable for demanding shielding applications, particularly through controlled ring-opening polymerization and tailored interfacial interactions in composite systems.

## Electromagnetic interference shielding in contemporary electronics

Modern electronics depend heavily on EMI shielding, a crucial technique that reduces the negative effects of unwanted electromagnetic signals that may interfere with the normal operation of electronic devices [12]. EMI results from external electromagnetic fields interacting with electronic circuits, which can lead to signal degradation, data loss, or device malfunction [13]. To ensure reliable system performance, effective EMI shielding involves the use of materials or structures designed to block, absorb, or deflect electromagnetic waves.

With the increasing complexity and miniaturization of electronic equipment, the importance of EMI shielding in modern electronics has become more pronounced. Contemporary electronic systems operate at high frequencies and are highly susceptible to electromagnetic interference, ranging from laptops and smartphones to advanced medical devices and communication networks [14]. As component density increases and high-speed data transmission becomes essential, the risk of EMI-related issues also rises. Consequently, robust shielding solutions are required to minimize interference and ensure compliance with stringent electromagnetic compatibility regulations [9].

Numerous EMI shielding materials and approaches have been developed to address these challenges. Conventional methods rely on conductive metals such as copper and aluminum, whose high electrical conductivity enables effective attenuation of electromagnetic waves [15]. Advances in materials science have led to the development of alternative shielding materials, including conductive polymers, composites, and coatings, which offer advantages such as flexibility, reduced weight, and ease of processing. These materials not only provide efficient shielding performance but also meet application-specific requirements, ranging from aerospace systems to portable consumer electronics.

In parallel, EMI shielding in clothing has emerged as an important area aimed at protecting users from potential health concerns associated with exposure to electromagnetic fields (EMFs) generated by modern electronic devices [16]. The growing adoption of wearable technologies has encouraged the integration of shielding materials into garments, such as metallic fiber-woven conductive fabrics and functional coatings. This approach enhances user comfort, supports compliance with safety standards, and provides practical benefits for individuals working in environments with elevated EMF exposure [16].

Despite the widespread use of conventional metallic and polymer-based EMI shielding materials, their limitations in terms of weight, corrosion resistance, thermal stability, and long-term reliability remain significant challenges in modern electronics [17,18]. These limitations have motivated increasing interest in advanced thermosetting systems such as benzoxazine-based materials. Owing to their high thermal stability, low dielectric constant, near-zero curing shrinkage, and structural design flexibility, benzoxazines represent a promising alternative platform for EMI shielding in contemporary electronic applications [19]. The following sections, therefore focus on benzoxazine chemistry, synthesis strategies, and structure–property relationships relevant to shielding performance.

## Challenges of EMI shielding

Electromagnetic interference shielding relies on fundamental mechanisms including reflection, absorption, and multiple internal scattering, which are governed by the electrical conductivity, dielectric properties, and magnetic permeability of the shielding material [6–8,20].

The shielding of electromagnetic interference (EMI) faces several challenges, particularly with the rapid advancement of wireless communication technologies and electronic devices [15]. One major challenge is the demand for lightweight and cost-effective materials that can maintain high shielding efficiency across a wide frequency range. Although traditional materials such as metals exhibit excellent electromagnetic wave reflection and conductivity, they are often heavy and susceptible to corrosion, making them less suitable for many modern applications. Another challenge involves developing materials capable of maintaining stable performance under varying environmental conditions, such as changes in temperature and humidity. In addition, achieving an optimal balance between mechanical flexibility and electrical conductivity remains a critical issue, especially for flexible and wearable electronic systems.

### Electrical conductivity

High electrical conductivity is essential for EMI shielding materials, as it enables effective reflection and absorption of electromagnetic waves [12]. This property is typically achieved using metals, carbon-based materials, or conductive polymers; however, balancing high conductivity with desirable mechanical properties can be challenging.

### Magnetic permeability

Materials with high magnetic permeability are particularly effective in absorbing low-frequency electromagnetic waves, which are commonly generated by electric motors and power transmission lines [14]. Ferrites and other magnetic materials are often employed for this purpose, although their incorporation can increase the complexity and weight of shielding system designs.

### Thermal stability

EMI shielding materials must retain their functional properties under thermal stress, especially in high-performance electronic applications where significant temperature fluctuations occur [21]. Materials that degrade or lose effectiveness at elevated temperatures can compromise device reliability and safety.

### Mechanical adaptability

Mechanical flexibility is a key requirement for shielding materials used in flexible circuits and wearable electronics [22]. Designing materials that can withstand bending or stretching without compromising their electromagnetic shielding performance remains a significant challenge, particularly for metal-based systems.

### Corrosion resistance and durability

Durability and resistance to environmental factors such as oxidation and corrosion are essential, particularly for outdoor and long-term applications [23]. Since many metals are prone to corrosion, EMI shielding systems often rely on protective coatings or alternative materials such as polymer- or composite-based systems to enhance service life.

## Benzoxazines: structure, thermal properties, and suitability for shielding applications

Benzoxazines, a class of thermosetting resins, are well known for their distinct chemical structure and advantageous thermal properties [24]. These characteristics make them suitable for a wide range of advanced applications, including protective coatings and electronic shielding. Benzoxazines exhibit inherent rigidity and stability due to their structural composition, which consists of a bicyclic oxazine ring fused to a benzene ring. This molecular architecture provides excellent resistance to heat, chemicals, and mechanical stress, which is essential for demanding industrial environments. In addition, benzoxazines display minimal curing shrinkage and outstanding dimensional stability, thereby reducing internal stresses in molded components. This behavior is particularly beneficial in applications such as electronic devices and aerospace components, where high durability and dimensional precision are required. Furthermore, benzoxazines demonstrate notable flame-retardant properties, enabling compliance with stringent safety standards without compromising performance.These properties originate from the ring-opening polymerization mechanism, which generates a highly cross-linked aromatic network upon curing.

Compared to conventional thermosetting resins, these features provide benzoxazines with superior dimensional stability and thermal endurance. Due to these inherent properties, benzoxazines are well suited for shielding applications. Their ability to form robust and thermally stable barriers enables effective protection against radiofrequency interference (RFI) and electromagnetic interference (EMI). This characteristic is increasingly important in modern electronic systems, where the widespread use of wireless technologies necessitates reliable shielding materials to minimize crosstalk and signal degradation.

## Synthesis methods of benzoxazines

**Scheme 1.**
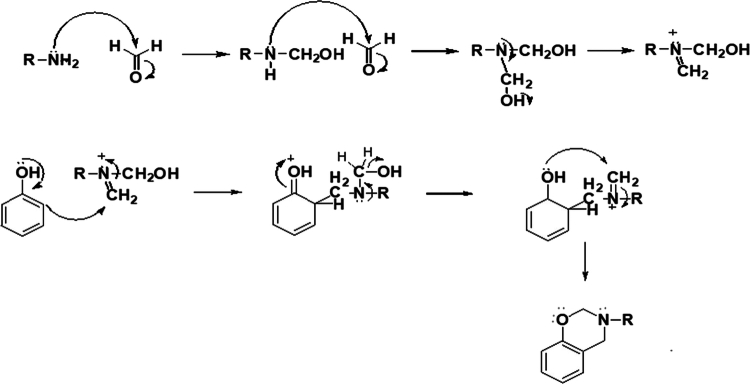
Common method for synthesizing benzoxazines and the associated reaction mechanism. This ﬁgure has been adapted/reproduced from Ref. [11] with permission from Elsevier.

The most common method for synthesizing benzoxazines is based on the condensation reaction between phenols, aldehydes, and amines, commonly referred to as the Mannich condensation reaction [11,25]. In this process, a nucleophilic attack occurs between the amine and the carbonyl carbon of the aldehyde, leading to the formation of an intermediate imine. Subsequently, the phenolic compound reacts with the imine intermediate, promoting condensation and facilitating the formation of the benzoxazine ring through a dehydration step. The final stage of the synthesis involves intramolecular cyclization, resulting in the formation of the benzoxazine heterocyclic structure. Overall, this sequence of reactions enables the efficient synthesis of benzoxazines from simple starting materials in a one-pot process (Scheme 1) [11].

This scheme illustrates the stepwise formation of benzoxazine monomers through a Mannich-type condensation reaction, followed by intramolecular ring closure. It is important to note that this synthesis stage yields benzoxazine monomers only. The thermally activated ring-opening polymerization and subsequent formation of highly cross-linked polybenzoxazine (PBz) networks occur in a separate curing step, as discussed in later sections. Such crosslinked PBz structures are ultimately responsible for the high thermal stability, low dielectric constant, and dimensional integrity required for effective electromagnetic interference (EMI) shielding in electronic and textile-based applications.

## Synthesis of some benzoxazine containing molecules

Investigation into high-performance polymers has consistently been a major focus for applications in demanding environments such as aerospace and electronic systems. Benzoxazines, noted for their excellent thermal stability, mechanical strength, and low moisture absorption, have played a significant role in the development of advanced thermosetting resins. Among these materials, bisphthalonitrile-containing benzoxazines (BPNBZ) have demonstrated superior thermal and oxidative stability, making them particularly suitable for high-temperature and high-reliability applications.

**Scheme 2.**
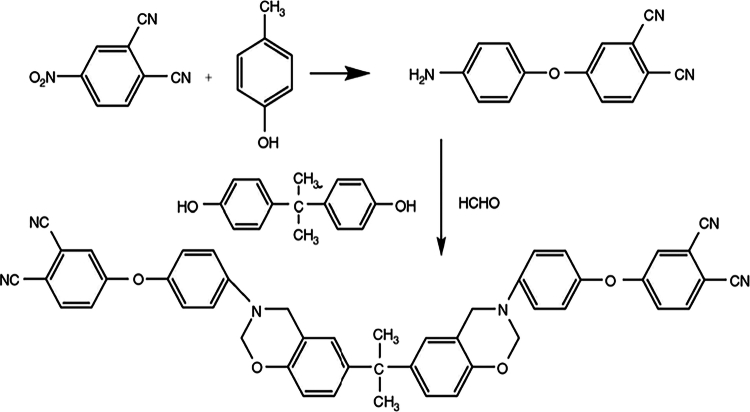
Synthesis and thermal polymerization behavior of bisphthalonitrile-containing benzoxazine (BPNBZ). This ﬁgure has been adapted/reproduced from Ref. [26], open access no permission required.

The synthesis of BPNBZ (Scheme 2), as reported by Cao et al. [26], involves the reaction of bisphenol A, 4-aminophenoxylphthalonitrile, and paraformaldehyde under reflux conditions at 110 °C. After solvent removal, the crude product is purified via dissolution in dimethylformamide (DMF) followed by precipitation in dilute sodium hydroxide solution. Spectroscopic analyses, including FTIR, ¹H NMR, and ¹³C NMR, confirmed the successful formation of the benzoxazine monomer containing both oxazine rings and phthalonitrile functionalities.

Upon thermal curing, the BPNBZ monomer undergoes a two-stage polymerization process. The first stage involves ring-opening polymerization of the benzoxazine moieties, while the second stage is associated with crosslinking reactions of the phthalonitrile groups, leading to the formation of a densely cross-linked polybenzoxazine (PBz) network.

This scheme depicts the thermal ring-opening polymerization of benzoxazine monomers, leading to the formation of highly cross-linked polybenzoxazine (PBz) networks. The ring-opening process generates phenolic hydroxyl groups and methylene bridges, which increase crosslink density and intermolecular interactions. These structural features are directly responsible for the high thermal stability, low dielectric constant, and mechanical robustness of PBz systems, all of which are critical parameters governing effective electromagnetic interference (EMI) shielding performance.

**Scheme 3.**
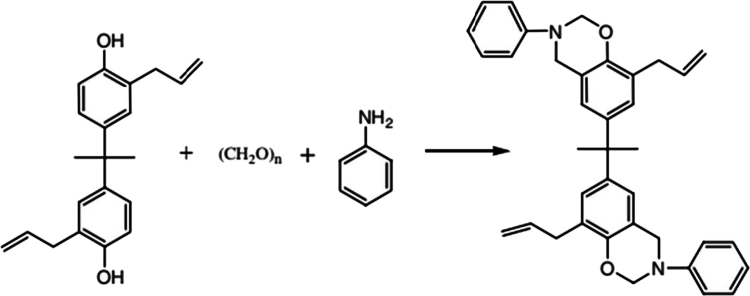
Synthesis of bis-allyl benzoxazines. This ﬁgure has been adapted/reproduced from Ref. [27] with permission from Elsevier

Kumar and colleagues [27] reported the synthesis of bis-allyl benzoxazines (Scheme 3), involving the preparation of a bis-benzoxazine monomer containing allyl groups through a solventless approach. The procedure employed 2, 2′-diallyl bisphenol A, paraformaldehyde, and aniline as key precursors. The synthesis began with mixing 2, 2′-diallyl bisphenol A and aniline, followed by the gradual addition of paraformaldehyde. The reaction mixture was subjected to a controlled temperature profile, starting with cooling in an ice bath and subsequently heating to 80 °C and then to 120 °C, where the final reaction proceeded for 2 h. The product was purified by solvent extraction and vacuum drying, yielding a reddish solid bis-allyl monomer with a melting point of 52–55 °C and an approximate yield of 60%.

This scheme illustrates the Mannich condensation mechanism involved in the synthesis of benzoxazine monomers from phenolic compounds, amines, and formaldehyde. The formation of the oxazine ring is a critical step, as it determines the subsequent ring-opening polymerization behavior and crosslinking density of the resulting polybenzoxazine network.

**Scheme 4.**
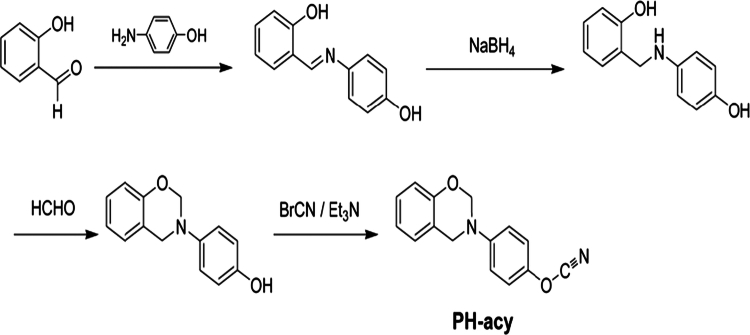
Synthesis of 2, 2′-bis (8-allyl-3-phenyl-3, 4-dihydro-2H-1, 3-benzoxazinyl) propane (Bz-allyl). This ﬁgure has been adapted/reproduced from Ref. [28] with permission from American Chemical Society.

In a related study, the synthesis of the benzoxazine monomer 2, 2′-bis(8-allyl-3-phenyl-3, 4-dihydro-2H-1, 3-benzoxazinyl)propane (Bz-allyl) was reported using a solvent-free method based on diallyl bisphenol A (DABA), aniline, and paraformaldehyde as the primary starting materials (Scheme 4) [28]. Diallyl bisphenol A was initially obtained by heating the diallyl ether of bisphenol A at 170 °C for 40 h, and its structure was confirmed by NMR analysis and hydroxyl value determination. Aniline was purified by vacuum distillation, while paraformaldehyde was used without further treatment. The resulting bis-allyl benzoxazine monomer was characterized using FTIR, ¹H NMR, and ¹³C NMR spectroscopy to confirm its chemical structure. This solvent-free synthetic route provides a straightforward and efficient approach for producing allyl-functionalized benzoxazine monomers suitable for high-performance polymer applications.

The introduction of allyl functionalities is expected to influence the curing behavior and network structure of the resulting polybenzoxazine systems, potentially enhancing thermal stability and mechanical integrity after polymerization. Such structural modifications may be advantageous for subsequent material design, where crosslink density and network rigidity are important structural parameters.

**Scheme 5.**
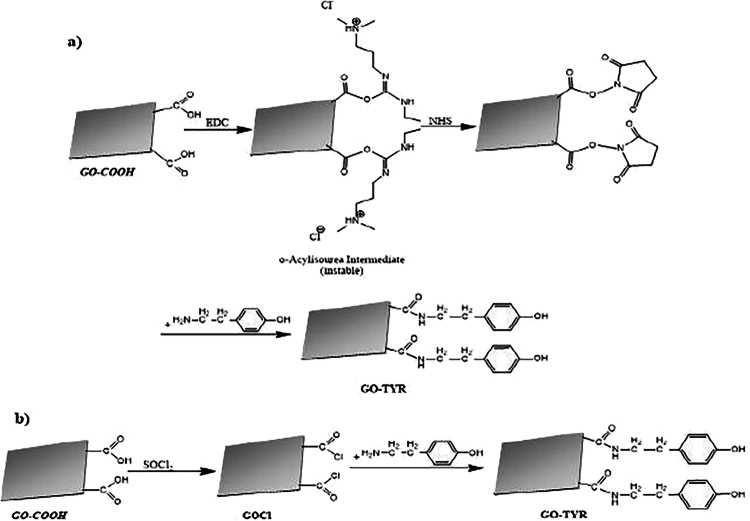
Synthesis of OH-functionalized graphene oxide obtained by: (a) EDC/NHS activation method, (b) chlorination with SOCl_2_. This ﬁgure has been adapted/reproduced from Ref. [29] with permission from Elsevier.

The research by Biru [29] describes the synthesis of benzoxazine-functionalized graphene oxide (GO-Bz) (Scheme 5a), which was initiated by the functionalization of graphene oxide (GO) containing carboxylic groups (GO–COOH). Two approaches were employed for this purpose: the EDC/NHS activation method and chlorination using thionyl chloride (SOCl₂) (Scheme 5a,b).

In the first approach, the carboxylic groups on graphene oxide were activated using 1-ethyl-3-(3-dimethylaminopropyl)carbodiimide (EDC) and N-hydroxysuccinimide (NHS). A phosphate buffer solution (PBS) at pH 5.5 was prepared, in which 5.42 mg of EDC was dissolved. Subsequently, 50 mg of GO–COOH and 12.07 mg of NHS were added, and the mixture was sonicated for 30 min in an ice bath. After adjusting the pH to 7.2 with PBS, 4.8 mg of tyramine was introduced, and the suspension was further sonicated for 90 min. The product was then filtered, washed with PBS (pH 5.5), and vacuum-dried for 48 h, yielding tyramine-functionalized graphene oxide (GO–TYR).

In the second approach, GO–COOH was chlorinated using thionyl chloride (SOCl₂) to generate acyl chloride groups on the graphene oxide surface. In this process, 100 mg of GO–COOH was refluxed with 60 mL of SOCl₂ and 0.5 mL of DMF at 76 °C for 24 h under a nitrogen atmosphere (Scheme 5b). The resulting acyl chloride-functionalized graphene oxide (GOCl) was obtained by removing excess solvent and SOCl₂ under reduced pressure. Subsequently, 9.6 mg of tyramine dissolved in tetrahydrofuran (THF) was added to GOCl, allowing the reaction between the acyl chloride groups and the amine groups of tyramine. The final product (GO–TYR) was washed six times with THF and dried overnight at 40 °C in a vacuum oven.

Scheme 5 presents the functionalization strategies employed to modify graphene oxide surfaces through the introduction of hydroxyl-rich moieties. Such surface modification is commonly used to improve the chemical compatibility of graphene oxide with polymeric matrices. In the context of benzoxazine-based systems, functionalized graphene oxide may facilitate improved dispersion and interfacial interactions during composite fabrication, which are important structural factors influencing the electromagnetic interference (EMI) shielding behavior of polymer nanocomposites.

**Scheme 6.**
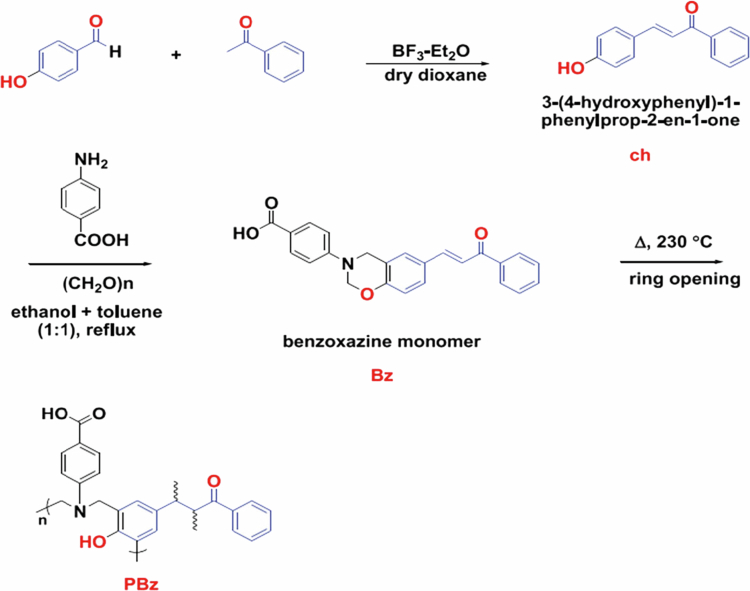
Synthesis of benzoxazine monomer (Bz) and polybenzoxazine (PBz). This illustrates the synthesis of the benzoxazine monomer and its subsequent thermal ring-opening polymerization to form PBz; the polymer structure is shown schematically. This ﬁgure has been adapted/reproduced from Ref. [30], open access no permission required.

Hamada et al. [30] reported the preparation of a benzoxazine monomer (Bz) using a 250 mL round-bottom flask. In this procedure, 4-aminobenzoic acid (1.37 g, 10 mmol) and paraformaldehyde (0.6 g, 20 mmol) were dissolved in 40 mL of an ethanol–toluene co-solvent (1:1, v/v) and stirred for 30 min. Subsequently, 1-(phenyl)-3-(4-hydroxyphenyl)prop-2-en-1-one (chalcone, 2.24 g, 10 mmol) was added, and the reaction mixture was refluxed at 90 °C under stirring for 48 h. After completion of the reaction, the mixture was cooled to room temperature. The resulting precipitate was dissolved in diethyl ether and washed with 150 mL of 1 M NaOH, yielding the benzoxazine monomer (Bz) with a yield of 2 g (Scheme 6).

The synthesized benzoxazine monomer was subsequently thermally polymerized by heating at 230 °C for 2 h in an oven to obtain polybenzoxazine (PBz). Characterization using FTIR, ¹H NMR, and TGA confirmed the formation of benzoxazine rings and demonstrated the enhanced thermal stability of the resulting PBz [30].

Scheme 6 illustrates the formation of the benzoxazine monomer followed by its thermal ring-opening polymerization into a cross-linked polybenzoxazine (PBz) network. The polymerization process results in a rigid aromatic structure with enhanced thermal stability. Such structural characteristics are generally desirable in high-performance polymer systems intended for demanding environments, where thermal resistance and dimensional stability are critical material requirements.

**Scheme 7.**
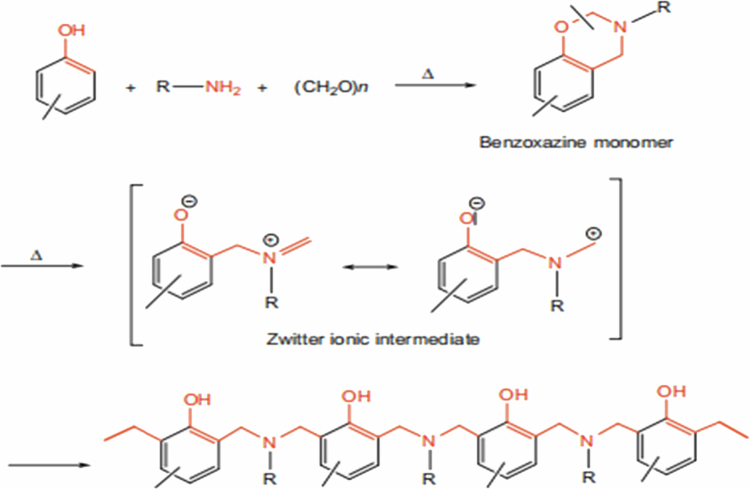
Synthesis of polybenzoxazine-clay nanocomposite. This ﬁgure has been adapted/reproduced from Ref. [31] with permission from Elsevier.

By incorporating montmorillonite (MMT) into a polybenzoxazine matrix, Agag and Akelah [31] reported the synthesis of a polybenzoxazine–clay nanocomposite (Scheme 7). In their study, a bisphenol A/aniline-based benzoxazine (BA-a) monomer was used as the precursor and subsequently polymerized to form the nanocomposite. The authors employed a melt-processing technique to achieve a uniform dispersion of organo-modified montmorillonite (OMMT) within the benzoxazine matrix. Initially, the BA-a monomer was melted, followed by the gradual addition of OMMT to obtain a homogeneous mixture. The resulting mixture was then transferred into a preheated mold and subjected to thermal curing to complete the polymerization and form the polybenzoxazine–clay nanocomposite. The incorporation of OMMMT as a reinforcing filler led to noticeable improvements in the thermal and mechanical properties of the resulting material [31].

Scheme 7 illustrates the synthesis of polybenzoxazine–clay nanocomposites, where layered clay platelets are incorporated into the polybenzoxazine matrix. The dispersion of organo-modified montmorillonite within the cross-linked PBz network leads to improved thermal stability and mechanical rigidity, as reported by Agag and Akelah [31]. The presence of well-dispersed clay layers enhances structural integrity and contributes to the overall robustness of the polymer matrix, which is desirable for advanced polymer systems subjected to thermal and mechanical stresses.

Abdous and coworkers [32] reported the synthesis of a boron carbide (B₄C)–filled polybenzoxazine nanocomposite using bisphenol A–based polybenzoxazine (BA-PBz) as the polymer matrix. The study aimed to enhance neutron shielding performance by exploiting the high neutron capture cross-section, hardness, and excellent thermal and chemical stability of boron-based compounds. Polybenzoxazine was selected to overcome the limitations of conventional phenolic resins due to its superior thermal stability and inherent flame-retardant characteristics.

The BA-Bz monomer was synthesized via a two-pot approach, followed by incorporation of boron carbide nanoparticles into the polymer matrix prior to thermal curing. To improve dispersion and interfacial adhesion, the B₄C nanoparticles were surface-modified using a silane coupling agent (KH-560). This surface functionalization promoted better compatibility between the inorganic filler and the polybenzoxazine matrix, leading to a more homogeneous composite structure.

Neutron shielding performance was evaluated at the Nuclear Research Reactor (NUR) in Algeria. The results demonstrated excellent shielding efficiency, particularly at a B₄C loading of 5 wt%, where the macroscopic cross-section reached *Σ* = 3.3878 cm⁻¹ and the screening ratio attained 97.78%. In addition to its high shielding efficiency, the nanocomposite exhibited good thermal stability, confirming its suitability for neutron and radiation shielding applications under harsh operating conditions.

**Scheme 8.**
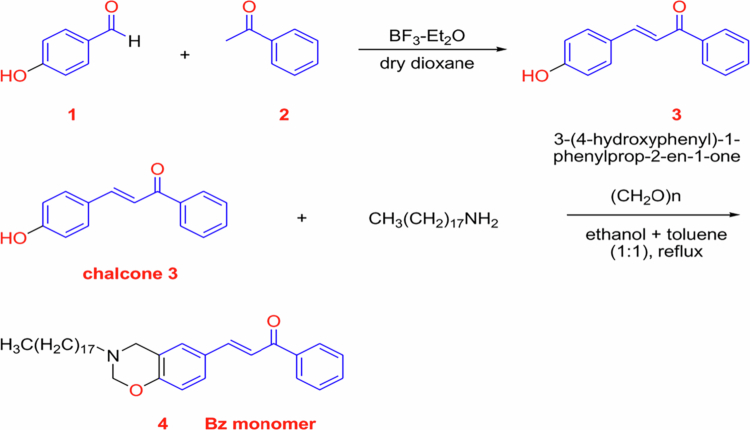
Synthesis of chalcone and chalcone-benzoxazine monomer. This ﬁgure has been adapted/reproduced from Ref. [33], open access no permission required.

Hamada et al. [33] reported the synthesis of a chalcone-derived benzoxazine–magnetite nanocomposite (Scheme 8). In their study, a benzoxazine monomer incorporating a chalcone moiety was synthesized via a Mannich condensation reaction involving 3-(4-hydroxyphenyl)-1-phenylprop-2-en-1-one, stearyl amine, and paraformaldehyde. Magnetite nanoparticles were subsequently incorporated into the benzoxazine system to obtain the corresponding nanocomposite.

The chalcone-containing benzoxazine underwent both photopolymerization and thermal curing to form cross-linked polybenzoxazine networks. Magnetite nanoparticles were added at different loadings, and their dispersion within the polymer matrix was assisted by sonication. The resulting materials exhibited enhanced thermal stability and magnetic properties, attributed to the homogeneous distribution of magnetite nanoparticles within the polybenzoxazine matrix.

The incorporation of aromatic chalcone structures and magnetic fillers into benzoxazine systems highlights a structural design strategy that may be relevant for multifunctional shielding materials, particularly where thermal stability and magnetic response are required alongside polymeric durability.

Scheme 8 illustrates the synthesis of chalcone and chalcone-functionalized benzoxazine monomers. The introduction of chalcone moieties expands the aromatic content and conjugation within the benzoxazine framework, which can influence the thermal behavior and network rigidity of the resulting polybenzoxazine after curing. Such structural features are relevant for material systems intended for multifunctional applications, where thermal stability and structural integrity are important design considerations.

**Figure 2.**
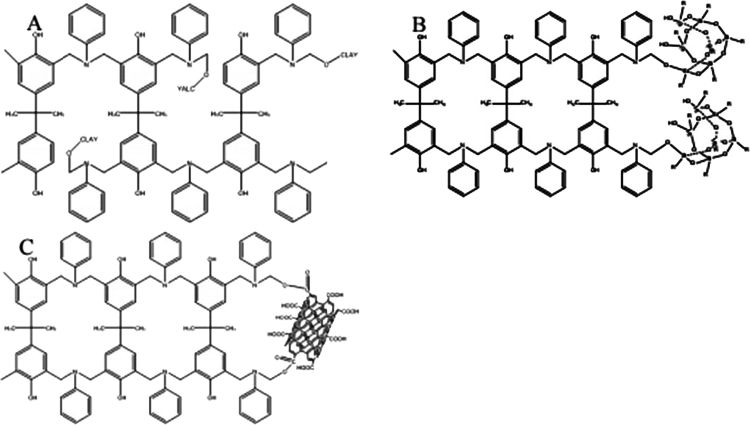
Bisphenol-A-based benzoxazine (BEN) nanocomposite (a–c). This ﬁgure has been adapted/reproduced from Ref. [34] with permission from John Wiley and Sons.

Bisphenol A–based benzoxazine (BEN) nanocomposites were synthesized by Yue et al. [34] using nanofillers such as clays, carbon nanotubes (CNTs), and polyhedral oligomeric silsesquioxane (POSS) (Figure 2a). The study examined the influence of these nanofillers on curing kinetics, network development, and the thermal stability of the benzoxazine matrix. The incorporation of these nanofillers aimed to enhance the thermal and mechanical properties of the BEN resin. The addition of POSS, CNTs, and clays significantly affected the curing behavior and thermal properties of the resulting nanocomposites. In particular, POSS incorporation led to a marked improvement in thermal stability, evidenced by increased char yield and prolonged degradation half-life under a nitrogen atmosphere.

Furthermore, multi-walled carbon nanotubes (MWCNTs) functionalized with hydroxyl (–OH) and carboxyl (–COOH) groups exhibited catalytic effects during the curing process, thereby enhancing the thermal degradation resistance of the composite. The results showed that the nanocomposite containing 4 wt% MWCNT–COOH achieved optimal thermal performance, with an increase of 286 °C in the half-life decomposition temperature and a 14 wt% rise in char yield at 800 °C (Figure 2b and c).

**Scheme 9.**
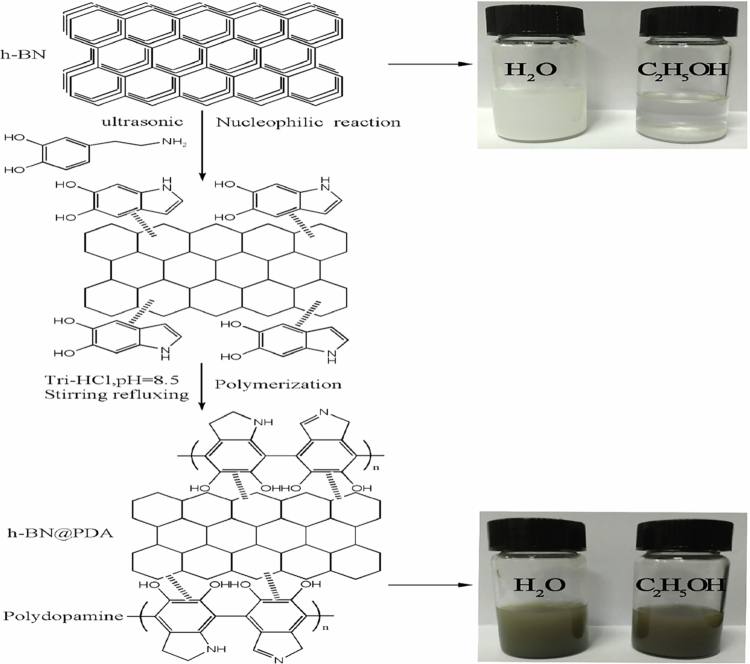
Synthesis of benzoxazine nanocomposite. This ﬁgure has been adapted/reproduced from Ref. [35] with permission from Elsevier.

Chen et al. [35] reported the synthesis of a benzoxazine nanocomposite designed to enhance thermal conductivity through the incorporation of non-covalently functionalized hexagonal boron nitride (h-BN) (Scheme 9). In this study, dopamine was used to coat the surface of h-BN in order to improve its dispersion and compatibility within the benzoxazine matrix. The non-covalent functionalization approach was employed to preserve the crystalline structure of h-BN while ensuring strong interfacial interactions between the polymer matrix and the nanofiller.

The resulting nanocomposites, containing different loadings of h-BN, exhibited significant improvements in thermal conductivity, particularly at higher filler contents. At an h-BN loading of 20 wt%, the dopamine-modified h-BN showed enhanced dispersion within the polymer matrix, leading to improved thermal pathways and reduced phonon scattering. In addition, the nanocomposites demonstrated improved thermal stability and mechanical properties due to the strong interfacial adhesion between h-BN and the polybenzoxazine matrix. The maximum thermal conductivity achieved was reported to be 0.71 W m⁻¹ K⁻¹, representing a substantial enhancement compared to unmodified composites. These results highlight the effectiveness of functionalized h-BN nanofillers in improving the thermal management performance of benzoxazine-based materials, making them suitable for electronic packaging applications.

Scheme 9 illustrates the synthesis route of benzoxazine-based nanocomposites via the incorporation of dopamine-modified h-BN nanosheets into the benzoxazine matrix prior to thermal curing. The dopamine functionalization promotes homogeneous dispersion of h-BN within the matrix and enhances interfacial interactions with the polymer network. Upon curing, a highly cross-linked polybenzoxazine structure is formed, which suppresses filler aggregation and contributes to improved thermal stability and mechanical integrity. These structural features are highly relevant for potential thermal management applications in electronic packaging systems.

Cha et al. [36] documented the synthesis of a benzoxazine nanocomposite incorporating functionalized multi-walled carbon nanotubes (MWCNTs) to enhance mechanical performance and environmental durability. The functionalization of MWCNTs was intended to improve their dispersion within the benzoxazine matrix and strengthen interfacial interactions with the polymer chains, ensuring a more uniform nanotube distribution and effective load transfer. As a result, the nanocomposite exhibited significant improvements in mechanical properties, including tensile strength and modulus, attributed to the strong interfacial bonding between the functionalized MWCNTs and the polymer matrix. In addition, enhanced thermal stability and resistance to environmental degradation were observed, indicating the suitability of the material for applications requiring durability under harsh conditions.

The incorporation of MWCNTs may also contribute to improved electrical characteristics, broadening the potential applicability of benzoxazine-based nanocomposites in advanced electronic and structural materials.

**Scheme 10.**
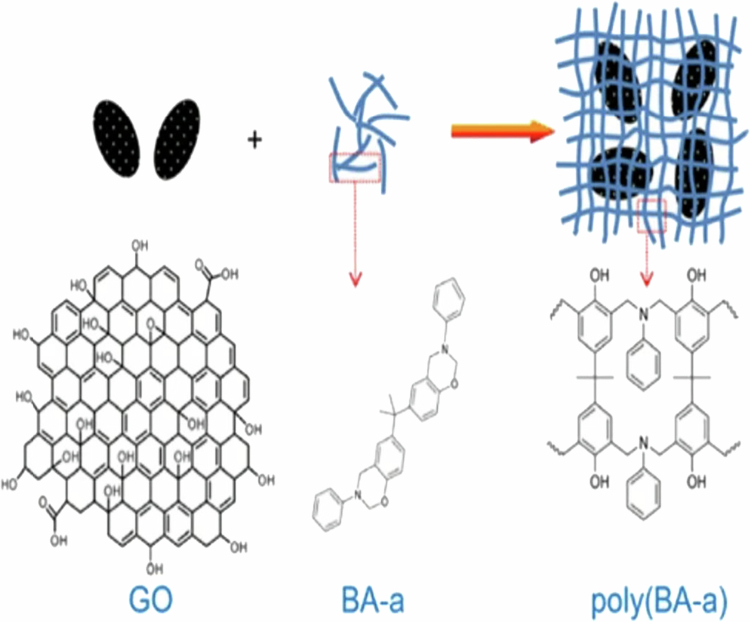
Benzoxazine nanocomposites, utilizing graphene oxide (GO). This ﬁgure has been adapted/reproduced from Ref. [37] with permission from Springer Nature.

Zhang et al. [37] documented the production of a reactive benzoxazine nanocomposite utilizing graphene oxide (GO) as a nanofiller to enhance polymerization behavior and thermal characteristics (Scheme 10). The oxygen functionalities on the GO surface were carefully regulated via hydrothermal reduction, allowing the authors to investigate their influence on the thermal polymerization of a bisphenol A–based benzoxazine monomer (BA-a). The study demonstrated that carboxyl and hydroxyl groups on GO accelerated the ring-opening polymerization of the benzoxazine monomer, leading to improved network formation. The resulting nanocomposites exhibited enhanced thermal conductivity and thermal stability, particularly at higher GO loadings. At a GO content of 6 wt%, the thermal conductivity increased from 0.27 W m⁻¹ K⁻¹ to 0.47 W m⁻¹ K⁻¹, corresponding to an enhancement of approximately 76%. In addition, increased degradation temperatures and char yield were observed, confirming the improved thermal stability of the nanocomposite. These findings highlight the important role of GO oxygen functionalities in tailoring the curing behavior and thermal performance of polybenzoxazine systems.

Scheme 10 illustrates the structural role of graphene oxide (GO) within benzoxazine-based nanocomposites. Owing to its two-dimensional layered morphology and oxygen-containing functional groups, GO can promote interfacial interactions and restrict polymer chain mobility within the cross-linked polybenzoxazine network. Such features are *potentially beneficial* for applications requiring enhanced thermal stability and dielectric response. Although the primary focus of this study is on thermal behavior, the described structural characteristics suggest that GO-filled benzoxazine systems may offer advantages for electromagnetic interference (EMI) shielding through absorption- and polarization-related mechanisms. The various modifications and advantages of these benzoxazine containing materials have been summarized in Table 1.

Key parameters influencing the synthesis process and the resulting properties of benzoxazine-based materials can be broadly classified into monomer structure, polymerization mechanism, curing conditions, and crosslinking density.

**Table 1.** Summary of reported benzoxazine-based systems, modifications, and their key advantages.

Phenol	Amine	Aldehyde	Modification	Advantages	Ref
Bisphenol-A	4-aminophenoxylphthalonitrile	Paraformaldehyde	Benzoxazine formation with phthalonitrile linkage	High thermal stability, good mechanical properties, electrical properties, and improved char yield at high temperatures, suitable for aerospace applications	[26]
Diallyl bisphenol-A	Aniline	Paraformaldehyde	Addition of allyl groups to benzoxazine monomer	Enhanced thermal stability, high cross-link density, elevated glass transition temperature (~300 °C), reduced amine loss during pyrolysis	[27]
Salicylaldehyde	4-aminophenol	Paraformaldehyde	Cyanate ester functionalization on benzoxazine	Lower polymerization temperature, high glass transition temperature, enhanced thermal stability, high char yield at elevated temperatures	[28]
Tyramine-derived hydroxyl groups on graphene oxide	Benzylamine	Formaldehyde	Benzoxazine rings grown on chemically modified graphene oxide	Strong covalent bonding between graphene sheets and polybenzoxazine chains, enhanced thermal and structural stability	[29]
Hydroxylated chalcone ((1-phenyl)-3-(4-hydroxyphenyl)prop-2-en-1-one)	4-aminobenzoic acid	Paraformaldehyde	Addition of magnetite nanoparticles	Improved thermal stability, low curing temperature (160 °C), enhanced magnetic properties with controlled magnetite incorporation	[30]
Bisphenol A	Aniline	Formaldehyde	Addition of organo-modified montmorillonite (OMMT)	Enhanced thermal and mechanical properties due to uniform dispersion of reinforcing clay nanoparticles	[31]
Bisphenol A	Aniline	Formaldehyde	Addition of boron carbide (B4C) nanoparticles with silane coupling	Improved neutron and gamma-ray shielding, enhanced thermal stability, effective radiation protection at 5 wt% B4C concentration	[32]
3-(4-hydroxyphenyl)-1-phenylprop-2-en-1-one (chalcone)	Stearyl amine	Paraformaldehyde	Integration of magnetite nanoparticles with sonication	Enhanced thermal stability and magnetic properties, uniform nanoparticle dispersion in the matrix	[33]
Bisphenol A	Not specified	Formaldehyde	Addition of nanofillers (POSS, CNTs, clays)	Enhanced thermal stability, improved char yield, increased thermal degradation resistance, catalytic effects from MWCNT-COOH	[34]
Not specified	Dopamine	Not specified	Incorporation of dopamine-functionalized hexagonal boron nitride (h-BN)	Enhanced thermal conductivity, improved thermal stability, superior dispersion and interfacial adhesion, reduced phonon scattering, suitable for electronic packaging	[35]
Not specified	Not specified	Not specified	Addition of functionalized multi-walled carbon nanotubes (MWCNTs)	Enhanced mechanical strength, improved tensile modulus, increased thermal stability, environmental durability, and electrical conductivity	[36]
Bisphenol A	Not specified	Not specified	Addition of graphene oxide (GO) with controlled oxygen functionalities	Enhanced thermal conductivity, improved thermal stability and char yield, accelerated ring-opening polymerization, improved polymer network development	[37]

### Monomer structure

The chemical nature of substituents on the phenolic ring plays a crucial role in determining the curing behavior, thermal stability, and polymerization characteristics of benzoxazine monomers. Electron-donating or electron-withdrawing groups can significantly influence ring-opening polymerization and the final network architecture. In addition, the introduction of functional groups such as allyl or cyanate ester moieties enables secondary reactions during curing, leading to higher crosslink density and enhanced thermal performance [25,28].

### Polymerization mechanism

Certain functionalized benzoxazine systems exhibit multi-stage polymerization behavior. As reported by Cao et al. [26] and Kumar et al. [27], polymerization may initially involve reactions of allyl or cyanate ester groups, followed by thermal ring-opening polymerization of the benzoxazine units. This sequential curing mechanism strongly affects the mechanical integrity and thermal resistance of the resulting polybenzoxazine network.

### Curing conditions

Curing temperature and duration directly influence the degree of polymerization and crosslinking density. Higher curing temperatures generally promote the formation of rigid and thermally stable networks, particularly in allyl- and cyanate-modified systems [25,28]. In some cases, the presence of reactive functional groups can lower the effective curing temperature or alter the curing exotherm [26].

### Crosslinking density and thermal–mechanical properties

Crosslinking density is a key factor governing the glass transition temperature (*T_g_*), thermal stability, and mechanical strength of polybenzoxazines. Polymers derived from allyl-functionalized monomers typically exhibit higher *T_g_* values, reaching approximately 300 °C due to additional crosslinking reactions [27]. Similarly, cyanate ester-containing systems demonstrate improved thermal stability and higher char yields, making them suitable for high-temperature applications [28]. Multifunctional monomers, such as bisphthalonitrile-based benzoxazines, further enhance network complexity and durability under extreme conditions [26].

Overall, careful control of monomer structure, polymerization pathway, curing conditions, and crosslinking density enables precise tuning of the thermal, mechanical, and chemical properties of polybenzoxazine materials for advanced engineering applications (Table 2).

**Table 2.** Preparation methods, key properties, and representative applications of benzoxazine-based materials reported in the literature.

Benzoxazine system	Preparation method	Key properties	Representative applications
BA-a (bisphenol A–aniline)	Mannich condensation followed by thermal curing	High thermal stability, low curing shrinkage, good mechanical strength	Electronic encapsulation, PCB substrates
Allyl-functional benzoxazines	Solventless Mannich reaction, stepwise thermal curing	High crosslink density, improved toughness, elevated *T_g_*	High-performance composites, aerospace materials
Phthalonitrile-containing benzoxazines	Mannich condensation with dual-stage polymerization	Excellent thermal and oxidative stability, high char yield	High-temperature aerospace and electronic components
Benzoxazine–graphene oxide composites	In situ mixing or surface-functionalized blending	Enhanced EMI shielding, improved thermal conductivity	EMI shielding films, electronic housings
Benzoxazine–CNT composites	Melt blending or solution-assisted dispersion	High electrical conductivity, improved mechanical reinforcement	EMI shielding coatings, flexible electronics
Benzoxazine-based textile coatings	Impregnation or coating followed by curing	Flame retardancy, low moisture absorption, durability	Protective clothing, functional textiles

Note: the reported properties correspond to polybenzoxazine networks obtained after thermal curing, unless otherwise stated.

## Applications of benzoxazines in electronics for EMI shielding

In modern electronic systems such as printed circuit boards (PCBs), integrated circuits (ICs), and wireless communication devices, effective EMI shielding is essential to ensure signal integrity and operational reliability, particularly at high frequencies [9,10,14–16,21,38–42].

Electromagnetic interference (EMI) is a growing concern in electronic devices due to the increasing density of electronic components and the widespread use of wireless communication technologies.

Benzoxazines, a class of thermosetting resins known for their excellent thermal stability, mechanical properties, and low moisture absorption, have gained significant attention for EMI shielding applications in electronics.

**Figure 3.**
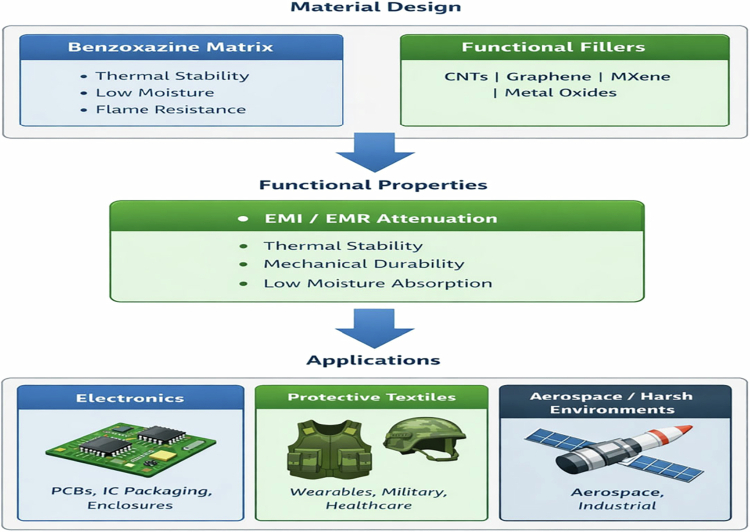
Conceptual overview illustrating the design strategy, filler integration, and application domains of benzoxazine-based materials for EMI shielding.

To summarize the application pathways and functional roles of benzoxazine-based materials in electronic shielding, a conceptual illustration is presented in Figure 3.

### Properties of benzoxazines ideal for EMI shielding

Benzoxazines possess several intrinsic properties that make them suitable candidates for electronic applications, particularly for EMI shielding:

#### Thermal stability

Benzoxazine-based materials exhibit high thermal stability, maintaining structural integrity and shielding effectiveness under the elevated temperatures typically encountered during electronic operation [43–45].

#### Low dielectric constant

A low dielectric constant minimizes signal loss, making benzoxazine composites suitable for high-frequency electronic applications where signal fidelity is critical [46].

#### Low moisture absorption

Moisture can significantly degrade shielding performance. Benzoxazine resins exhibit low moisture uptake, ensuring stable shielding performance even under humid environmental conditions [45].

#### Excellent adhesion

Benzoxazines demonstrate strong adhesion to a wide range of substrates, which is particularly important for EMI shielding coatings in electronic systems [47].

### Specific applications in electronic devices

#### EMI shielding in printed circuit boards (PCBs)

Printed circuit boards (PCBs) are fundamental components of modern electronic devices, ranging from consumer electronics to automotive systems. EMI can disrupt PCB functionality, leading to signal interference, data corruption, or component failure. Benzoxazine-based composites have been increasingly explored for PCB applications due to their favorable dielectric properties and effective EMI shielding capability. In PCB structures, benzoxazines are commonly used as part of multilayer composite matrices or as surface coatings. Their ability to form highly cross-linked networks upon curing enables efficient attenuation of electromagnetic waves, thereby protecting sensitive electronic components [48].

#### Encapsulation and coatings for integrated circuits (ICs)

Integrated circuits are particularly susceptible to EMI due to their high integration density. Benzoxazine-based encapsulants and coatings have been applied to shield ICs from both internal and external electromagnetic interference. The low dielectric constant of benzoxazine materials allows encapsulated ICs to operate with minimal signal loss while maintaining effective EMI protection [49].

#### EMI shielding in wireless communication devices

With the rapid expansion of wireless communication devices, including smartphones, routers, and Internet of Things (IoT) systems, EMI shielding has become increasingly critical. Benzoxazine composites, especially those incorporating conductive fillers such as carbon nanotubes (CNTs) or graphene, have been developed to form lightweight and efficient shielding layers. These materials can be integrated into device casings, where they both suppress incoming EMI and reduce electromagnetic emissions from the devices themselves [50].

### Case studies and examples of benzoxazine implementation

#### EMI shielding in aerospace electronics

Aerospace electronics require materials capable of withstanding extreme environmental conditions while maintaining reliable electronic performance. Benzoxazine-based composites have been implemented as EMI shielding materials in aerospace applications due to their excellent thermal stability, flame retardancy, and mechanical strength. In satellite communication systems, benzoxazine composites have been applied as shielding layers to protect onboard electronics from space radiation and EMI. Compared with conventional shielding materials, these composites demonstrated reduced signal interference while offering significant weight savings, which are crucial for aerospace applications [36].

#### EMI shielding in automotive electronics

Modern automotive systems increasingly rely on electronic control units (ECUs), which are highly susceptible to EMI due to their proximity to high-power components. Benzoxazine composites have been utilized to fabricate EMI shielding enclosures for ECUs, providing both electromagnetic protection and improved thermal management. For example, benzoxazine resins reinforced with carbon fibers have been reported to enhance EMI shielding effectiveness while improving heat dissipation, enabling reliable ECU operation under elevated temperature conditions [51].

#### Wearable electronics

Wearable electronic devices require effective EMI shielding solutions that are lightweight and minimally intrusive. Benzoxazine-based composites, particularly when combined with conductive fillers such as silver nanoparticles or carbon-based materials, have been investigated for wearable applications due to their flexibility, low density, and high shielding effectiveness. In one reported case, graphene-doped benzoxazine composites were used in smartwatch casings, providing effective EMI shielding while maintaining a slim profile and wearer comfort. The use of these materials resulted in reduced signal interference from surrounding electronic devices, leading to improved device performance [52].

### Advances in benzoxazine-based EMI shielding composites

Recent developments in benzoxazine-based composites have led to materials with significantly enhanced EMI shielding performance. Hybrid benzoxazine composites incorporating conductive fillers such as graphene, carbon nanotubes, and metal nanoparticles have demonstrated improved shielding effectiveness due to synergistic organic–inorganic interactions. Benzoxazine–graphene composites, for example, combine high mechanical strength with excellent EMI shielding effectiveness, particularly in the GHz frequency range, making them promising candidates for next-generation communication technologies such as 5G systems [53].

Similarly, benzoxazine–CNT composites have exhibited high electrical conductivity and effective EMI shielding performance when applied as coatings on flexible electronic substrates. These characteristics make them especially attractive for flexible electronics and wearable devices, where conventional metallic shielding materials are often unsuitable due to rigidity or weight constraints [54].

Overall, benzoxazines have emerged as highly promising materials for EMI shielding applications in electronics. Their combination of thermal stability, low moisture absorption, and favorable dielectric properties enables their use across a wide range of electronic devices, including PCBs, ICs, wireless communication systems, and wearable electronics. The continued development of benzoxazine-based composites with conductive fillers is expected to further expand their role in advanced electronic systems, ensuring reliable performance as device miniaturization and integration continue to advance [55].

## Applications of benzoxazines in clothing and textiles for electromagnetic radiation (EMR) shielding

With the rapid increase in electronic devices and the widespread adoption of wireless communication technologies, exposure to electromagnetic radiation (EMR) has become an increasing concern. Protective clothing, particularly for individuals working in high-EMR environments such as medical, military, and industrial settings, is being actively developed using materials capable of providing effective EMR shielding. Benzoxazines, a class of high-performance thermosetting resins, have emerged as promising candidates in this field due to their unique combination of thermal stability, mechanical strength, and dielectric properties. When incorporated into textiles, benzoxazine-based materials offer several advantages over traditional shielding materials for EMR protection.

Recent studies have increasingly focused on functional and protective textiles designed to mitigate electromagnetic radiation exposure in medical, military, and wearable applications [16,41,42,56,57].

### Properties of benzoxazines in EMR shielding for textiles

Recent studies have highlighted significant progress in functional and protective textiles for electromagnetic shielding, particularly through the use of sustainable and bio-based materials. Cellulose- and biomass-derived textile composites have demonstrated high EMI shielding effectiveness while maintaining flexibility, lightweight characteristics, and environmental sustainability [58,59].

Benzoxazines exhibit several intrinsic properties that make them highly suitable for textile-based EMR shielding applications:

#### Thermal and flame resistance

Benzoxazine-based materials demonstrate excellent thermal stability and inherent flame retardancy, making them well suited for protective clothing used in environments involving elevated temperatures and EMR exposure [60].

#### Low dielectric constant

A low dielectric constant is a critical requirement for EMR shielding materials, as it minimizes signal interference. Benzoxazines exhibit low dielectric values, enabling effective reduction of electromagnetic radiation impact on the wearer [12].

#### Durability and mechanical strength

Benzoxazines are characterized by high mechanical strength and structural integrity, enabling textile-based shielding materials to withstand mechanical stress and repeated use without significant degradation in EMR shielding performance [61–63].

#### Chemical resistance

The chemical resistance of benzoxazine resins ensures that treated textiles maintain their shielding properties even when exposed to oils, solvents, or harsh chemical environments [64].

In addition, the low moisture uptake and excellent chemical resistance of benzoxazine-based systems contribute to the long-term stability of textile shielding materials, particularly under humid or chemically aggressive conditions, where conventional polymer systems often suffer performance degradation [65,66].

### Specific applications in protective clothing

#### Military and defense clothing

Military personnel are frequently exposed to high levels of electromagnetic radiation, particularly in environments involving radar systems, communication equipment, and electronic warfare technologies. Benzoxazine-based textiles are being investigated for military applications due to their effective EMR shielding capability combined with thermal and chemical resistance. Benzoxazine composites incorporating conductive fillers such as metal nanoparticles or carbon-based materials can be integrated into military uniforms, providing lightweight, flexible, and durable EMR shielding while maintaining protection against fire and chemical agents. The inherent durability of benzoxazine-based textiles enables them to withstand demanding operational conditions without degradation in performance [67].

#### Healthcare protective gear

In medical environments, especially those utilizing high-frequency equipment such as magnetic resonance imaging (MRI) systems and radiation-emitting devices, protective apparel is essential to reduce electromagnetic field exposure to healthcare professionals. Benzoxazine-based textiles have been engineered to provide effective EMR shielding while preserving flexibility and wearer comfort. When incorporated into fabrics, benzoxazine resins form protective barriers that reduce electromagnetic radiation exposure while allowing breathability and moisture-wicking properties, ensuring comfort during prolonged use [68].

#### Industrial and civilian protective wear

Workers in industries such as telecommunications, power generation, and electronics manufacturing are often exposed to elevated levels of electromagnetic radiation. Benzoxazine-based protective garments offer effective shielding against prolonged EMR exposure while maintaining mechanical robustness. In these applications, benzoxazine resins are typically combined with conductive fillers to produce flexible and resilient fabrics suitable for garments such as jackets, gloves, and aprons. These textiles provide both EMR shielding and thermal resistance, making them suitable for environments where electromagnetic radiation and heat are present simultaneously [69].

### Benefits of using benzoxazines over traditional materials

The use of benzoxazines in EMR-shielding textiles provides several advantages compared with conventional materials such as metal-based fabrics, carbon fibers, and traditional polymer systems.

#### Lightweight and flexible nature

One of the primary limitations of conventional EMR shielding materials, particularly metal-based textiles, is their high weight and limited flexibility. While metallic fabrics offer effective shielding, they can be heavy and uncomfortable. Benzoxazine-based composites are significantly lighter and more flexible, enabling the design of protective clothing that offers both comfort and effective EMR shielding. When combined with conductive fillers such as carbon nanotubes (CNTs) or graphene, benzoxazine-based textiles can be incorporated into stretchable or form-fitting garments, which is particularly advantageous in medical and military applications.

#### Enhanced durability and wear resistance

Traditional EMR shielding materials, including metallic fabrics and carbon fibers, may degrade over time due to exposure to moisture, heat, or chemicals. Benzoxazine-based textiles exhibit enhanced resistance to abrasion, chemical exposure, and environmental degradation. The high cross-linking density of cured benzoxazine resins contributes to long-term structural integrity, reducing the need for frequent replacement and extending service life compared with conventional alternatives [70].

#### Fire retardancy and heat resistance

Many conventional EMR shielding polymers lack adequate fire retardancy and thermal resistance. Benzoxazine-based textiles inherently exhibit superior fire resistance due to their aromatic structure and high char yield, which promote the formation of protective barriers during combustion. This dual functionality—EMR shielding combined with fire and heat resistance—makes benzoxazine-based textiles particularly attractive for high-risk industrial and military environments [71].

#### Moisture and chemical resistance

Benzoxazine-based textiles demonstrate superior resistance to moisture and chemical exposure compared with traditional shielding materials. Unlike metal-based fabrics, which may corrode under humid or chemically aggressive conditions, benzoxazine-treated textiles retain their mechanical and shielding performance. The low moisture absorption of benzoxazines ensures long-term stability of EMR shielding effectiveness, even in outdoor or harsh environmental conditions [72].

Compared to conventional metallic or carbon-based shielding textiles, advanced polymer-based systems—including benzoxazine-compatible matrices—offer superior processability, durability, and long-term stability. Recent reviews emphasize the growing role of polymer-reinforced functional textiles in medical, military, and industrial protective clothing [73,74].

Recent studies have investigated alternative textile-based electromagnetic radiation (EMR) shielding materials, including metal oxide nanoparticles and metal–organic frameworks (MOFs).

Metal oxides such as Fe₃O₄ and TiO₂ have been incorporated into fabrics to introduce magnetic and dielectric losses, contributing to electromagnetic radiation (EMR) attenuation while maintaining reasonable flexibility [57,75,76]. However, achieving high shielding effectiveness often requires relatively high filler loadings, which may negatively affect fabric comfort, breathability, and long-term durability.

MOFs have also attracted increasing attention in functional and protective textiles due to their tunable porosity and multifunctional characteristics [57,77,78]. Nevertheless, their application in EMR shielding clothing remains limited by challenges related to electrical conductivity, mechanical robustness, and large-scale processability.

In comparison, benzoxazine-based textile composites—particularly when hybridized with conductive fillers such as graphene or carbon nanotubes—offer a more balanced combination of shielding effectiveness, thermal stability, durability, and textile compatibility. This makes benzoxazine systems more suitable for long-term protective clothing applications than many nanoparticle- or MOF-based textile systems [76,79].

Despite the promising performance of benzoxazine-based textiles, their shielding efficiency is often optimized through hybridization with conductive fillers, highlighting the importance of composite design rather than resin chemistry alone. This observation is consistent with recent advances in multifunctional protective textiles reported in the literature.

### Advances in benzoxazine-based textiles for EMR shielding

**Figure 4.**
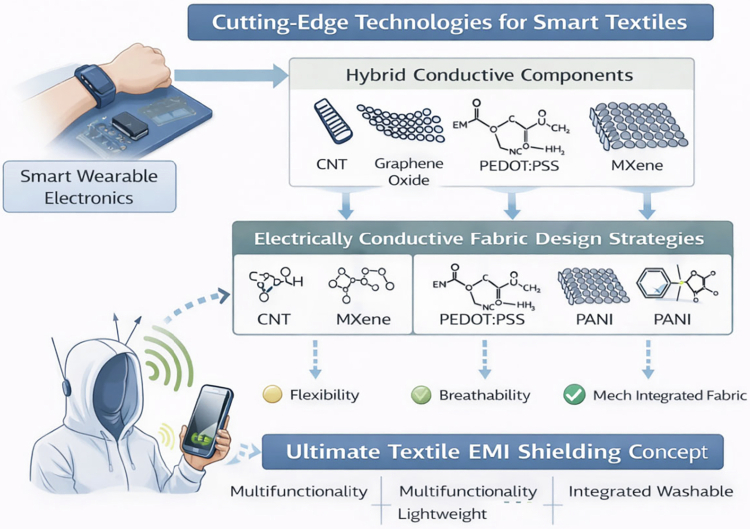
Conceptual roadmap for next-generation textile and wearable EMI shielding technologies, illustrating the progression from current smart wearable electronics toward multifunctional, lightweight, and washable EMI-shielding textiles through synergistic integration of advanced conductive fillers and fabric design strategies.

Recent research efforts have focused on enhancing both the shielding effectiveness and mechanical performance of benzoxazine-based textiles. A key advancement involves the incorporation of conductive fillers such as graphene, carbon nanotubes, and metal nanoparticles into the benzoxazine matrix. Benzoxazine–graphene composites have demonstrated high EMR shielding efficiency across a broad frequency range while maintaining flexibility and lightweight characteristics. These materials are being actively investigated for next-generation protective clothing that requires a combination of durability, flexibility, and high shielding performance [80]. Similarly, benzoxazine–CNT composites exhibit high electrical conductivity and mechanical strength, making them suitable for applications where long-term durability and EMR shielding are required (Table 3) (Figure 4).

**Table 3.** Summarizes representative benzoxazine-based systems reported in literature, highlighting preparation strategies, key properties, and application areas relevant to functional and protective materials.

Benzoxazine system	Preparation method	Key properties	Applications	Representative refs
Bio-based benzoxazine composites	Natural phenols/bio-based feedstocks, Mannich condensation	Sustainable, high thermal stability, low shrinkage	Functional composites, coatings	[11,81,82]
PBz conductive composites	Monomer design + conductive fillers (CNTs, graphene)	Improved electrical conductivity, thermal stability	EMI shielding, electronics	[36,61,83]
Benzoxazine/GO/CNT systems	In situ mixing/functionalized GO	Enhanced dielectric, thermal & electrical properties	Flexible EMI shielding	[29,36,47]
Standard benzoxazine resins	Mannich condensation + thermal curing	High Tg, low shrinkage, flame retardancy	Coatings, structural parts	[43,63,65]
Phosphorus-modified PBz systems	Chemical modification/flame-retardant design	Enhanced flame retardancy & heat resistance	Protective coatings, electronics	[64,84]

## Durability and long-term performance of benzoxazine-based shielding materials

Benzoxazine-based shielding materials have attracted significant interest due to their superior mechanical and thermal properties, along with their adaptability for applications requiring electromagnetic radiation (EMR) protection. Durability and long-term performance are critical factors in evaluating their suitability for protective garments, electronic systems, and related applications. These materials must not only provide effective shielding but also retain their properties over extended periods, even under harsh environmental conditions such as humidity, extreme temperatures, and ultraviolet (UV) exposure.

### Long-term durability of benzoxazine-based shielding materials

Benzoxazines exhibit several intrinsic properties that contribute to their outstanding durability:

#### High cross-linking density

Upon curing, benzoxazine resins form a densely cross-linked network, enhancing mechanical strength, thermal stability, and chemical resistance. This robust structure enables resistance to physical degradation, making benzoxazine materials suitable for applications requiring long-term service life [85].

#### Resistance to chemical degradation

Benzoxazine-based composites exhibit strong resistance to a wide range of chemicals, including acids, bases, oils, and solvents. Unlike some conventional materials that may deteriorate upon prolonged chemical exposure, benzoxazines maintain their protective performance and mechanical integrity over time [86].

#### Low water absorption

A key factor influencing long-term shielding performance is susceptibility to moisture uptake. Benzoxazine-based materials demonstrate low water absorption, which is essential for preserving mechanical integrity and shielding effectiveness, particularly in humid environments [86].

### Stability under environmental conditions

Benzoxazine-based materials must withstand various environmental stressors, including humidity, temperature fluctuations, and UV exposure, which often degrade conventional shielding materials. Benzoxazines exhibit excellent stability under these conditions, ensuring sustained performance over time.

#### Humidity and moisture resistance

Moisture absorption can significantly reduce the mechanical and shielding performance of many polymer-based materials. Benzoxazine-based shielding systems display superior resistance to moisture uptake due to their inherently low water absorption. This makes them suitable for environments with high humidity, such as outdoor and industrial settings [6]. Experimental studies have shown that benzoxazine composites subjected to high-humidity conditions exhibit negligible changes in mechanical strength and dielectric properties, maintaining their EMR shielding effectiveness. This behavior represents a clear advantage over conventional materials such as polyimides or epoxy-based composites, which may degrade over time in moist environments [10].

#### Thermal stability

Thermal stability is essential for long-term shielding performance, particularly in high-temperature environments such as aerospace, automotive, and industrial applications. Benzoxazines exhibit exceptional thermal stability, enabling them to maintain shielding performance at elevated temperatures [15].

Benzoxazine-based systems typically exhibit glass transition temperatures (*T_g_*) higher than those of conventional polymers such as epoxies. As a result, they retain structural integrity and functionality at temperatures where other materials may soften or degrade. This characteristic is especially critical in electronic and aerospace applications, where sustained EMR shielding at elevated temperatures is required [15].

In thermal cycling studies simulating aircraft operating conditions, benzoxazine composites retained structural integrity and EMR shielding performance after repeated cycles, demonstrating excellent thermal durability [87].

#### UV resistance and weatherability

Ultraviolet (UV) radiation can degrade polymer chains, leading to embrittlement, discoloration, and reduced shielding performance over time [88]. Benzoxazine-based materials exhibit enhanced resistance to UV-induced degradation compared with conventional polymers such as epoxies and polyesters. This resistance is primarily attributed to the aromatic structure of benzoxazines, which provides improved photostability [88].

Studies evaluating benzoxazine composites under prolonged UV exposure have reported minimal surface degradation and sustained shielding efficiency, confirming their suitability for outdoor and long-term applications [89].

### Case studies on long-term performance

#### Aerospace applications

In aerospace environments, materials are subjected to extreme temperature variations, high humidity, and UV exposure. Benzoxazine-based composites evaluated under these conditions have demonstrated excellent long-term durability. In one case study involving satellite components requiring EMR shielding, benzoxazine materials were exposed to space radiation, thermal cycling, and UV radiation. After extended service periods, the materials showed negligible degradation while maintaining shielding effectiveness and structural integrity, highlighting their suitability for aerospace applications [90].

#### Automotive electronics

Automotive electronic components, such as engine control units (ECUs) and infotainment systems, operate under elevated temperatures and continuous vibration. Benzoxazine-based composites tested for ECU enclosures demonstrated superior thermal stability, moisture resistance, and mechanical durability compared with conventional epoxy materials. These properties ensured sustained shielding effectiveness throughout the operational lifespan of the vehicle.

#### Industrial protective clothing

Protective garments used in high-EMR industrial environments require both effective shielding and long-term durability. Benzoxazine-based textiles reinforced with conductive fillers such as graphene or carbon nanotubes have demonstrated excellent resistance to moisture, heat, and chemical exposure. Long-term evaluations revealed that these textiles retained flexibility, EMR shielding performance, and mechanical integrity after prolonged industrial use, outperforming conventional materials that showed noticeable degradation over time.

### Advances in enhancing long-term performance

Recent advances in benzoxazine-based composites have focused on improving long-term durability under harsh environmental conditions. Hybrid systems combining benzoxazine resins with conductive fillers such as carbon nanotubes, graphene, and metal nanoparticles have been shown to enhance mechanical performance and EMR shielding effectiveness [91]. These hybrid composites also demonstrate improved resistance to thermal degradation, UV exposure, and moisture absorption, thereby extending service life in demanding environments. In addition, advanced curing strategies have been reported to increase cross-linking density in benzoxazine networks, further enhancing durability and environmental stability.

## Future directions and challenges in benzoxazine-based shielding materials

**Figure 5.**
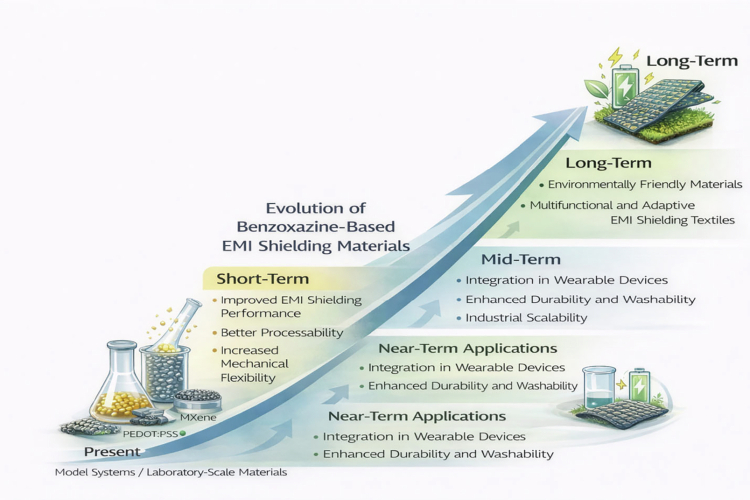
Conceptual roadmap of future development directions for benzoxazine-based EMI shielding materials (short-, mid-, and long-term).

Benzoxazine-based materials are acknowledged for their exceptional qualities, especially in electromagnetic radiation (EMR) shielding. As technical requirements escalate and the need for sophisticated materials in high-performance applications intensifies, researchers are investigating novel avenues for the development of benzoxazine composites. Nonetheless, several obstacles related to performance enhancement, cost-effectiveness, and scalability must be resolved for benzoxazines to completely achieve their potential in commercial and industrial applications. This section delineates future research trajectories and developments in benzoxazine-based shielding materials, along with the hurdles that must be surmounted to expand their application (Figure 5).

## Future research directions and potential innovations

### Development of hybrid composites

An interesting research field is the creation of hybrid composites that integrate benzoxazines with other materials to improve their performance. The integration of conductive fillers like graphene, carbon nanotubes (CNTs), and metallic nanoparticles into benzoxazine matrices can markedly enhance their electromagnetic interference (EMI) shielding performance, mechanical strength, and thermal conductivity. Hybrid composites provide a means to develop high-performance materials that are lightweight, flexible, and resilient to severe weather conditions. Research on graphene-doped benzoxazine composites indicates that low graphene loadings can significantly improve the electrical conductivity and shielding efficacy of the materials, rendering them appropriate for applications necessitating substantial electromagnetic radiation protection. The integration of CNTs has been shown to enhance the electrical and mechanical properties of benzoxazines, resulting in composites with enhanced durability and flexibility for applications such as protective apparel or electronic devices [91].

### Nanostructured benzoxazine-based materials

Nanotechnology presents promising prospects for the enhancement of benzoxazine-based shielding materials. Nanostructured materials can enhance material properties at the molecular level, allowing for improved control over the thermal, mechanical, and dielectric aspects of composites [46]. Nanomaterials, including metal oxides, nanoclays, and carbon-based substances, can be integrated into benzoxazine matrices to enhance performance in electromagnetic interference shielding, thermal resistance, and flame retardancy.

The advancement of self-healing nanocomposites signifies a prospective innovation for enhancing the durability and dependability of benzoxazine-based materials in demanding conditions. Researchers seek to develop materials with enhanced long-term durability by including nanostructured components capable of autonomously repairing minor flaws.

### Low-temperature curing technologies

Future research will primarily concentrate on the advancement of low-temperature curing processes for benzoxazine resins. Conventional benzoxazine curing typically necessitates elevated temperatures, which may restrict their utilization, especially in electronics and textiles where thermal sensitivity is a significant issue. Researchers aim to lower the curing temperature by investigating alternate curing agents or catalysts, while maintaining the material's mechanical strength and shielding efficacy.

Reducing the curing temperature may enhance the application of benzoxazines in sectors such as flexible electronics and wearable devices, where heat-sensitive components are prevalent. This will decrease energy consumption and manufacturing expenses related to benzoxazine synthesis, enhancing their appeal for large-scale industrial applications [81].

### Bio-based and sustainable benzoxazine resins

Due to escalating environmental concerns, there is a rising interest in the advancement of bio-based benzoxazine resins. Conventional benzoxazine synthesis depends on petroleum-derived feedstocks, prompting apprehensions regarding sustainability and ecological consequences. Future research aims to synthesize benzoxazines from renewable resources, including plant-derived oils or lignin, thereby diminishing the carbon footprint of these materials.

Bio-based benzoxazines offer environmental advantages while demonstrating mechanical and thermal qualities equivalent to those of petroleum-based alternatives. Research in this domain focuses on enhancing the scalability and performance of bio-based benzoxazines to establish them as feasible alternatives for electromagnetic radiation shielding applications.

### Functionalization for enhanced shielding

Another pathway for future innovation entails the functionalization of benzoxazine resins to customize their properties for particular uses. Functionalization may entail altering the chemical structure of the benzoxazine monomer or integrating additives to improve particular qualities such as electrical conductivity, flexibility, or flame retardancy. The incorporation of ionic liquids or metallic nanoparticles into benzoxazine matrices enhances their electrical conductivity and electromagnetic interference shielding efficacy, rendering them more appropriate for high-frequency electronic applications [92]. Additional investigation into these modifications will enable the tailoring of benzoxazine-based materials to fulfill the distinct requirements of various industries.

### Challenges and areas for improvement

Despite the advantages offered by benzoxazine-based materials, several challenges remain that need to be addressed to improve their performance and enable large-scale adoption.

#### High processing temperatures and costs

A primary challenge of benzoxazine materials is the elevated curing temperature required for cross-linking. Curing temperatures for benzoxazines sometimes surpass 200 °C, hence restricting their use in applications involving heat-sensitive materials [93]. Elevated processing temperatures can increase energy consumption and manufacturing expenses, rendering large-scale production less economically viable. Research efforts aim to create low-temperature curing systems that preserve the advantageous characteristics of benzoxazines while minimizing the energy required for processing. This may entail employing innovative catalysts, alternative cross-linking agents, or hybrid systems that integrate benzoxazines with other thermosetting polymers that cure at reduced temperatures [94].

#### Mechanical toughness and flexibility

Benzoxazines are recognized for their superior thermal and dielectric properties; however, mechanical toughness and flexibility may present challenges in specific applications. In flexible electronics or wearable devices, the brittleness of certain benzoxazine-based composites may restrict their use [11]. Enhancing the mechanical properties of benzoxazines, while preserving their electromagnetic radiation shielding efficacy, remains a critical focus for future research. Researchers are investigating the integration of flexible fillers, such as elastomers, and the use of toughening agents to enhance the impact resistance and flexibility of benzoxazine composites.

#### Scalability and cost-effectiveness

Despite the numerous performance advantages of benzoxazine resins, their elevated production costs and scalability challenges continue to pose substantial obstacles to widespread commercial application. The cost of raw materials, coupled with the energy-intensive curing process, renders benzoxazines less competitive than alternative thermosetting polymers, such as epoxies or phenolics, in large-scale applications [54].

Future research should concentrate on cost-reduction strategies to enhance the accessibility of benzoxazine materials [95]. This may include the development of more efficient manufacturing techniques, the utilization of lower-cost raw materials, or innovations in bio-based benzoxazines that may significantly reduce dependence on petroleum-derived inputs. Moreover, improvements in curing processes, such as rapid-curing systems or ambient-temperature curing techniques, may substantially lower manufacturing costs and enhance the economic viability of benzoxazines [96].

#### Environmental impact and recycling

Benzoxazine-based materials exhibit superior thermal stability and durability compared to many other polymers; however, their environmental implications, particularly regarding disposal and recycling, remain a concern. Conventional benzoxazine resins are not readily recyclable, and their long-term environmental persistence raises sustainability issues [81].

Researchers are investigating sustainable benzoxazines derived from renewable resources and strategies to improve the recyclability of benzoxazine-based materials. The development of recyclable benzoxazine composites or biodegradable benzoxazines may reduce the environmental impact of these materials, increasing their attractiveness for industries that prioritize sustainability [97].

**Figure 6.**
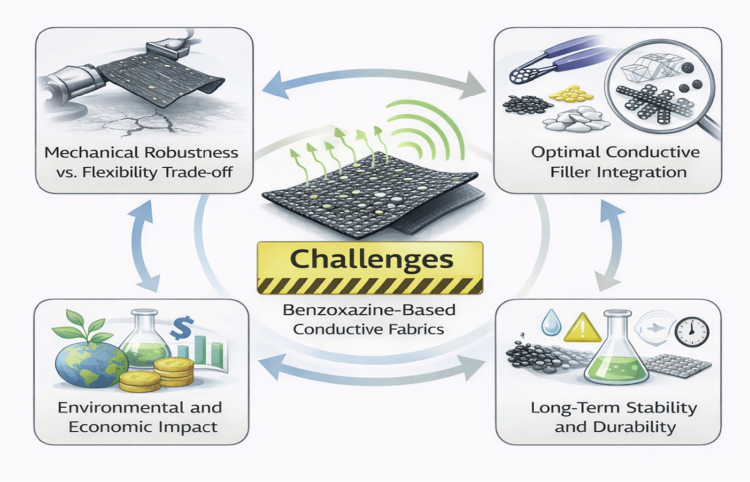
Conceptual overview of the main challenges in developing benzoxazine-based EMI shielding textiles, highlighting the interrelated requirements of mechanical robustness, conductive filler integration, long-term stability and durability, and environmental and economic considerations.

To visually summarize the key challenges associated with benzoxazine-based EMI shielding textiles, a conceptual overview is presented in Figure 6.

## Benzoxazine supramolecular structural divergence

Benzoxazine-based materials are renowned for their exceptional thermal, mechanical, and electromagnetic shielding characteristics; however, their potential involvement in supramolecular chemistry remains relatively underexplored, which presents opportunities for advanced material design rather than conventional synthesis routes. Supramolecular chemistry pertains to the examination of molecular assemblages created via non-covalent interactions, including hydrogen bonding, van der Waals forces, π–π interactions, and host–guest chemistry. When applied to benzoxazine systems, these interactions can result in structural divergence, enabling the generation of various material morphologies and properties through controlled molecular organization rather than covalent modification. This section examines the utilization of supramolecular interactions in benzoxazine systems to develop novel material capabilities applicable to diverse high-performance domains.

### Supramolecular chemistry in benzoxazine systems

**Figure 7.**
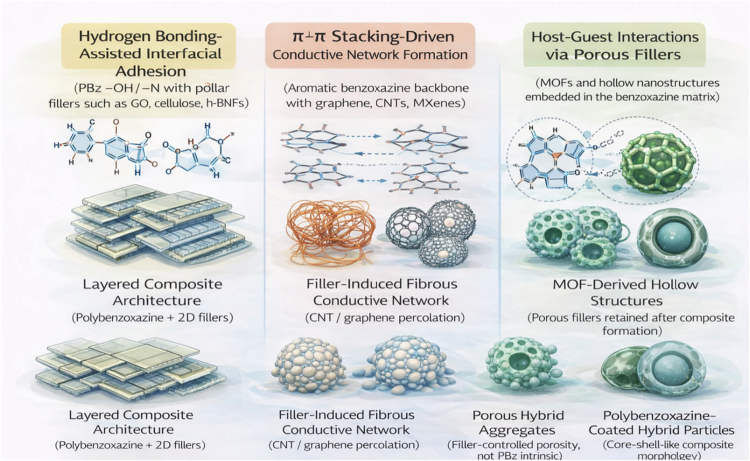
Schematic illustration of dominant intermolecular interactions (hydrogen bonding, π–π stacking, and host–guest interactions via porous fillers) governing the formation of hierarchical structures in benzoxazine-based nanocomposites for EMI shielding and functional textile applications.

Benzoxazines possess a distinctive molecular structure that facilitates supramolecular self-assembly. The benzene ring, oxazine core, and their associated functional groups can engage in non-covalent interactions, enabling the formation of diverse supramolecular architectures. These assemblies can be tuned to achieve specific material properties relevant to applications in electronics, textiles, and coatings (Figure 7).

#### Hydrogen bonding

Hydrogen bonding represents a primary non-covalent interaction governing the self-assembly of benzoxazine-based systems. The nitrogen atom in the oxazine ring and the hydroxyl groups generated during ring-opening polymerization can act as both hydrogen bond donors and acceptors [66]. These interactions promote organized supramolecular networks, directly influencing mechanical strength, thermal stability, and dielectric behavior. When benzoxazine monomers are functionalized with additional hydrogen-bonding moieties (e.g., carboxyl or amine groups), enhanced molecular ordering and phase organization can be achieved, which is advantageous for applications requiring controlled morphology, such as EMI shielding and flame-retardant textiles.

#### π–π stacking interactions

The aromatic rings within benzoxazine monomers enable π–π stacking interactions, contributing to the stability and alignment of supramolecular assemblies. These interactions are particularly important for generating layered or stacked morphologies, which can enhance thermal conductivity and mechanical robustness in benzoxazine-based composites [98]. For example, incorporation of aromatic nanofillers such as graphene promotes strong π–π interactions between the filler and benzoxazine matrix, yielding structured nanocomposites with improved electrical conductivity and EMI shielding efficiency. Such supramolecular reinforcement provides a clear advantage over purely covalent composite designs, particularly in electronic applications where thermal management and shielding must coexist.

#### Van der Waals forces and host–guest chemistry

In addition to hydrogen bonding and π–π stacking, van der Waals forces contribute to the stabilization of supramolecular benzoxazine assemblies, particularly in multicomponent systems containing fillers or dopants.

Furthermore, host–guest chemistry can expand the design space of supramolecular benzoxazine materials. In such systems, benzoxazine derivatives may act as host molecules capable of interacting with guest species such as cyclodextrins or calixarenes, forming inclusion complexes with tailored mechanical or thermal behavior [1]. These host–guest architectures are particularly attractive for stimuli-responsive materials, enabling adaptive responses to temperature, pH, or electromagnetic fields.

### Supramolecular structural divergence in benzoxazine materials

Controlled manipulation of non-covalent interactions in benzoxazine systems enables the formation of structurally diverse supramolecular architectures. This structural divergence manifests in the development of nanofibers, lamellar structures, vesicles, and other morphologies, each offering distinct functional advantages for high-performance applications.

#### Nanofibers and nanotubes

Benzoxazine monomers can self-assemble into nanofibers or nanotubes through cooperative hydrogen bonding and π–π interactions. These structures exhibit high surface-area-to-volume ratios, making them attractive for catalysis, filtration, sensing, and advanced textile applications [99]. Benzoxazine-based nanofibers also demonstrate excellent thermal and chemical stability, enabling their use in harsh environments. When combined with nanofillers such as carbon nanotubes or metal oxides, these supramolecular fibers exhibit enhanced electrical conductivity and EMI shielding effectiveness, expanding their applicability in smart fabrics and flexible electronics.

#### Lamellar and layered structures

Supramolecular interactions can also drive the formation of lamellar or multilayer benzoxazine architectures. These layered systems are highly desirable for multifunctional coatings and membranes due to their combined mechanical strength, thermal resistance, and selective permeability [100]. From an EMI shielding perspective, multilayer architectures provide multiple internal reflection pathways, significantly improving shielding efficiency. Benzoxazine–graphene oxide multilayer composites exemplify this approach, where π–π interactions promote ordered filler alignment, resulting in lightweight materials with enhanced thermal conductivity and shielding performance suitable for aerospace and automotive applications [101].

#### Vesicles and capsules

Benzoxazine monomers are also capable of forming vesicular or capsule-like structures through carefully controlled supramolecular interactions. These architectures are relevant for encapsulation, controlled release, and self-healing applications. In self-healing coatings, benzoxazine-based vesicles can release healing agents upon mechanical damage, restoring structural integrity and maintaining shielding performance. Such behavior is particularly valuable in long-term EMR shielding applications, where microcracks or defects can severely compromise material performance.

### Applications of supramolecular benzoxazine structures

The supramolecular structural diversity achievable in benzoxazine systems enables a wide range of advanced applications [102]:

**Electronics and photonics:** nanostructured benzoxazine assemblies, including nanofibers and layered composites, offer combined EMI shielding and thermal regulation for compact electronic and photonic devices.

**Protective textiles**: supramolecular benzoxazine fibers and vesicles enable the design of lightweight yet durable fabrics capable of shielding against EMR, heat, and chemical exposure.

**Catalysis and filtration:** the high surface area and tunable chemistry of benzoxazine nanofibers make them suitable for catalytic supports and selective filtration membranes.

### Challenges and future directions

Despite the promise of supramolecular benzoxazine systems, several challenges must be addressed:

#### Control of supramolecular interactions

Achieving precise control over non-covalent interactions remains challenging due to their sensitivity to environmental factors such as temperature, humidity, and pH. Developing predictive design strategies for supramolecular assembly is essential to ensure reproducible material performance.

#### Scalability of production

While supramolecular benzoxazine materials are readily produced at laboratory scale, industrial-scale fabrication remains challenging due to cost, process complexity, and structural uniformity. Advances in self-assembly control and scalable processing techniques will be critical for commercial adoption.

#### Environmental stability

Although benzoxazines exhibit inherent thermal and mechanical robustness, the long-term stability of their supramolecular architectures under extreme conditions (UV radiation, high temperature, or chemical exposure) requires further investigation. Maintaining supramolecular integrity over extended service lifetimes is essential, particularly for applications in electronics and protective clothing.

## Advanced shielding and conductivity in benzoxazine-based nanocomposites: The role of MWCNTs, carbon fibers, MXenes, nanoclay, and epoxy

Benzoxazine-based nanocomposites have attracted increasing attention due to their exceptional thermal stability, mechanical strength, flame resistance, and lightweight characteristics, rendering them suitable for a wide range of high-performance applications. However, pristine benzoxazine systems often exhibit limited electrical conductivity, which constrains their direct use in electromagnetic interference (EMI) shielding applications. Consequently, the incorporation of functional nanofillers—including multi-walled carbon nanotubes (MWCNTs), carbon fibers, Ti₃C₂ (MXenes), nanoclay, and epoxy—has become a critical strategy to overcome these intrinsic limitations. These nanofillers impart additional functionalities, such as enhanced electrical conductivity, mechanical reinforcement, and improved thermal and chemical resistance to benzoxazine matrices [83]. Through careful filler selection and interfacial design, benzoxazine nanocomposites can be tailored for advanced applications in aerospace, electronics, and structural engineering.

### MWCNTs as nanofillers in benzoxazine nanocomposites

Multi-walled carbon nanotubes (MWCNTs) are widely recognized for their outstanding mechanical, electrical, and thermal properties. When incorporated into benzoxazine matrices, MWCNTs significantly enhance composite performance by forming conductive percolation networks, owing to their high aspect ratio, large specific surface area, and excellent intrinsic conductivity. These networks directly contribute to improved electrical conductivity, EMI shielding effectiveness, and mechanical reinforcement.

MWCNTs have been effectively employed as nanoscale reinforcements in hybrid benzoxazine/epoxy/carbon fiber (CF) systems, where they act not only as conductive bridges but also as stress-transfer agents within the matrix. In such composites, MWCNTs enhance structural integrity and facilitate charge dissipation, leading to improved EMI shielding efficiency [103]. The synergistic interaction between MWCNTs and carbon fibers results in superior interfacial adhesion, reduced porosity, and enhanced thermal stability, collectively improving the composite's durability and shielding performance [104].

Furthermore, the conductive nature of MWCNTs enables their application in electrostatic discharge (ESD) protection and high-frequency EMI shielding materials [23]. Benzoxazine/MWCNT nanocomposites have demonstrated enhanced dielectric properties and effective shielding performance, particularly in the GHz frequency range, making them suitable for modern electronic and communication devices.

### Carbon fibers in benzoxazine composites

Carbon fibers (CFs) are valued for their high tensile strength, low density, and excellent thermal stability. When used as reinforcements in benzoxazine matrices, carbon fibers significantly improve stiffness and strength-to-weight ratios, which are critical for aerospace and automotive applications [105].

In addition to mechanical reinforcement, carbon fibers contribute to thermal and electrical conductivity, particularly when combined with secondary conductive fillers such as MWCNTs or metallic nanoparticles [106]. This hybrid reinforcement strategy enables the development of multifunctional composites that simultaneously provide mechanical robustness and EMI shielding capability.

A key challenge associated with carbon fiber composites is achieving strong interfacial adhesion between the fibers and the polymer matrix. Benzoxazine resins exhibit inherently favorable interfacial compatibility with carbon fibers due to their unique curing chemistry and low shrinkage behavior, reducing the risk of delamination and mechanical degradation. As a result, benzoxazine–carbon fiber composites demonstrate enhanced thermal stability, flame resistance, and dimensional integrity [107].

### Ti₃C₂ (MXene) as nanofillers for enhanced conductivity and shielding

MXenes, particularly Ti₃C₂, have emerged as promising two-dimensional nanomaterials due to their exceptional electrical conductivity, large surface area, and layered morphology [108]. These characteristics make them highly effective nanofillers for benzoxazine-based composites aimed at EMI shielding and electrical conductivity enhancement.

When incorporated into benzoxazine matrices, MXenes facilitate the formation of interconnected conductive pathways, thereby significantly improving EMI shielding efficiency. Their tunable surface chemistry enables strong interfacial interactions with the polymer matrix, leading to improved dispersion and mechanical integrity [21]. Additionally, Ti₃C₂ MXenes contribute to mechanical reinforcement while maintaining lightweight characteristics.

MXene-filled benzoxazine composites have demonstrated superior shielding effectiveness in microwave and radiofrequency (RF) ranges, highlighting their suitability for electronic packaging, antenna housings, and high-frequency shielding applications.

### Nanoclay in benzoxazine nanocomposites

Nanoclays, such as montmorillonite (MMT), are extensively investigated as nanofillers due to their ability to enhance thermal stability, mechanical performance, and flame retardancy in polymer systems [109]. In benzoxazine-based composites, nanoclays act as reinforcing and barrier agents, increasing stiffness, reducing flammability, and improving gas and heat resistance [110].

Nanoclays possess layered silicate structures with high aspect ratios, allowing effective intercalation or exfoliation within the benzoxazine matrix when properly dispersed [111]. Such hierarchical structures significantly restrict polymer chain mobility and heat transfer, leading to improved thermal and mechanical properties. Consequently, nanoclay-reinforced benzoxazine composites exhibit enhanced flame retardancy and reduced smoke generation, making them suitable for transportation, construction, and fire-resistant applications.

### Epoxy as a matrix modifier in benzoxazine nanocomposites

Epoxy resins are widely known for their excellent mechanical strength, chemical resistance, and adhesive performance. Blending benzoxazine with epoxy results in hybrid thermosetting systems that combine the advantages of both resins, including improved toughness, thermal stability, and dimensional control [112].

The presence of epoxy enhances the processability of benzoxazine systems, enabling easier molding and fabrication of complex structures. When combined with conductive fillers such as MWCNTs or MXenes, epoxy-containing benzoxazine composites support the formation of continuous conductive networks, thereby enhancing electrical conductivity and EMI shielding performance.

The synergistic interaction between benzoxazine and epoxy improves impact resistance and environmental durability while preserving the inherent flame retardancy and thermal stability of benzoxazines [101]. As a result, benzoxazine/epoxy nanocomposites are well suited for demanding applications in aerospace, automotive, and electronic components (Table 4).

**Table 4.** Summarizes and compares the effects of different nanofillers on the electrical conductivity, EMI shielding performance, thermal properties, and targeted applications of benzoxazine-based nanocomposites reported in the literature.

Nanofiller type	Main role in benzoxazine matrix	Key enhanced properties	Typical shielding/conductivity contribution	Representative applications	References
MWCNTs	Formation of conductive percolation networks	Electrical conductivity, mechanical strength, EMI shielding	High EMI shielding effectiveness (especially GHz range) due to interconnected conductive pathways	EMI shielding coatings, flexible electronics, aerospace components	[23,36,103,104]
Carbon fibers (CFs)	Structural reinforcement and load transfer	High stiffness, strength-to-weight ratio, thermal stability	Moderate EMI shielding, enhanced when combined with CNTs or metals	Aerospace panels, automotive electronics enclosures	[105–107]
MXenes (Ti₃C₂)	Highly conductive 2D filler with layered structure	Electrical conductivity, EMI shielding, thermal dissipation	Strong absorption-dominant EMI shielding in RF and microwave ranges	Electronic packaging, RF shielding layers	[21,108]
Nanoclay (MMT)	Barrier formation and flame-retardant reinforcement	Thermal stability, flame retardancy, mechanical stiffness	Indirect EMI shielding via multilayer barrier and dielectric loss	Fire-resistant electronics housings, protective coatings	[31,109–111]
Graphene/GO	Conductive filler and polymerization catalyst	EMI shielding, thermal conductivity, curing enhancement	High EMI shielding efficiency via reflection and absorption mechanisms	Lightweight EMI shields, wearable electronics	[29,37,80]
Metal nanoparticles	Conductive and reflective filler	Electrical conductivity, EMR shielding	Strong reflection-based EMR shielding, limited flexibility	Protective clothing, radiation shielding textiles	[67,92]
Epoxy (hybrid matrix)	Toughening and processability enhancement	Mechanical toughness, dimensional stability	Indirect EMI shielding when combined with conductive fillers	Structural electronics, aerospace composites	[101,112]

This comparative analysis highlights that the shielding efficiency and conductivity of benzoxazine-based systems are strongly governed by filler dimensionality, dispersion quality, and interfacial interactions rather than filler type alone.

## Environmental impact and sustainability of benzoxazine composites

The increasing need for sophisticated materials in shielding and electronics necessitates addressing the environmental impact of benzoxazine-based composites. Conventional benzoxazines are primarily synthesized from petroleum-derived raw materials, which raises concerns regarding long-term sustainability and environmental footprint. Initiatives are in progress to create bio-based benzoxazines derived from renewable resources such as plant oils, lignin, and other biomass derivatives [113]. These bio-based alternatives aim to reduce dependence on fossil resources while maintaining comparable mechanical and thermal performance to conventional benzoxazines.

Furthermore, the disposal and recycling of thermosetting resins such as benzoxazines remain challenging due to their highly cross-linked network structures, which limit their reprocessability and recyclability [114]. Investigations into biodegradable benzoxazines and recycling strategies, including chemical recycling and controlled degradation, are still at an early stage; nonetheless, encouraging outcomes have emerged in the development of self-healing benzoxazine systems, which may extend material service life and consequently reduce material waste [88]. Moreover, the adoption of more sustainable synthesis routes, such as solvent-free processes or energy-efficient catalytic systems, represents a promising strategy to further lower the environmental impact associated with benzoxazine production [115].

## Comparative efficacy of benzoxazines versus conventional shielding materials

Benzoxazines are distinguished by their exceptional thermal stability, low dielectric constant, and excellent mechanical strength, rendering them highly competitive alternatives to conventional shielding materials such as metals, conductive polymers, and carbon-based composites [116]. Metals such as copper and aluminum have long been regarded as benchmark materials for electromagnetic interference (EMI) shielding owing to their superior electrical conductivity. However, their high density, susceptibility to corrosion, and lack of flexibility significantly limit their suitability for lightweight, flexible, or wearable applications [117].

Benzoxazines offer superior thermal stability and inherent flame retardancy, which are critical for high-temperature applications in the aerospace and automotive sectors. Moreover, their low moisture absorption represents a major advantage over traditional polymeric materials, which often suffer from performance degradation in humid environments [118]. In addition, benzoxazines exhibit enhanced chemical resistance, contributing to long-term durability under harsh operating conditions where conventional materials may fail [82].

Numerous studies have evaluated the performance of benzoxazine-based shielding materials in comparison with conventional metals and polymers, particularly in applications requiring lightweight, multifunctional, and durable shielding solutions, such as aerospace and advanced electronics.

### Shielding against space radiation

Benzoxazine-based composites exhibit strong potential for space applications owing to their lightweight nature and radiation-shielding capability. A study assessing shielding efficiency under galactic cosmic ray (GCR) conditions compared benzoxazine/ultra-high-molecular-weight polyethylene (UHMWPE) composites with conventional materials such as aluminum and epoxy-based composites. The results demonstrated that benzoxazine/UHMWPE systems achieved lower equivalent radiation doses than aluminum, highlighting their improved radiation-shielding performance. This enhancement was attributed to the higher hydrogen content of the composite system, which improves neutron attenuation while simultaneously reducing overall structural weight—an essential requirement for spacecraft materials [45].

### Thermal and mechanical stability in electronics

Benzoxazine-based materials are increasingly employed in electronic applications where both EMI shielding and thermal stability are critical. Studies indicate that benzoxazine composites reinforced with nanofillers such as carbon nanotubes (CNTs) or graphene oxide (GO) outperform conventional epoxy-based systems in terms of thermal resistance and mechanical stability. The improved performance arises from strong interfacial interactions between the nanofillers and the inherently heat-resistant benzoxazine matrix. In particular, benzoxazine/CNT composites exhibit higher thermal conductivity and lower degradation rates than epoxy/CNT counterparts, making them more suitable for electronic enclosures and EMI shielding under elevated temperature conditions [11].

### Multifunctional structural components

Carbon fiber-reinforced benzoxazine composites demonstrate clear advantages in multifunctional structural applications. When incorporated into sandwich structures with UHMWPE cores, these materials provide a balanced combination of radiation shielding, mechanical strength, and reduced weight. Comparative investigations have shown that benzoxazine-based sandwich composites surpass aluminum and traditional carbon fiber–epoxy laminates in terms of specific strength and radiation-shielding efficiency. Additionally, their resistance to thermal and chemical degradation extends component service life, particularly in environments where metallic materials are prone to corrosion or mechanical failure [119].

Overall, these comparative studies underscore the distinct advantages of benzoxazine-based shielding materials over conventional metal and polymer systems, particularly in applications demanding lightweight design, durability, and multifunctionality. Through tailored nanofiller incorporation and matrix design, benzoxazine composites not only compete with but, in many cases, exceed the performance limits of traditional shielding materials.

Conventional EMI shielding materials such as metals, epoxy-based composites, and carbon fiber systems have been widely used; however, their performance is often limited by weight, corrosion, or processing constraints [5,10,82,116–119].

To facilitate a direct comparison with conventional shielding materials, a comparative overview of benzoxazine-based shielding materials and conventional systems is summarized in Table 5.

The comparison is intended to provide a qualitative assessment of key performance attributes, including thermal stability, mechanical durability, flexibility, and shielding applicability, based on representative studies discussed in the preceding sections. This overview highlights general trends rather than exhaustive numerical benchmarking and is included to facilitate a clear conceptual comparison between material classes.

**Table 5.** Comparison of benzoxazine-based shielding materials with conventional shielding systems.

Material system	Density/weight	EMI/radiation shielding performance	Thermal stability	Mechanical durability	Flexibility/wearability	Key limitations	Typical applications
Metals (Cu, Al)	High	Excellent EMI shielding due to high conductivity	Moderate–high	Good, but prone to fatigue	Very limited	Heavy weight, corrosion, poor flexibility	Electronic enclosures, rigid housings
Epoxy-based composites	Moderate	Moderate (improved with fillers)	Moderate	Good	Limited	Moisture sensitivity, thermal degradation	PCBs, coatings
Carbon fiber composites	Low–moderate	Moderate EMI, weak radiation shielding	High	Excellent	Limited	Brittleness, processing complexity	Aerospace structures
Conductive polymer systems	Low	Moderate	Low–moderate	Moderate	Good	Poor thermal resistance	Flexible electronics
Benzoxazine-based composites	Low	High (especially with CNTs/GO/MXenes)	High	Excellent	Good (textile-compatible)	High curing temperature, cost	Electronics, aerospace, protective textiles
Benzoxazine/UHMWPE systems	Low	High radiation shielding	High	High	Moderate	Processing complexity	Space and aerospace shielding

The comparison is based on representative studies discussed in Section 14.

## Recent developments in curing methods for benzoxazines

Conventional benzoxazine resins necessitate elevated curing temperatures, frequently surpassing 200 °C, which limits their applicability on heat-sensitive substrates such as flexible electronics and textile-based systems [120]. Recent developments have therefore focused on low-temperature curing strategies aimed at expanding the processing window of benzoxazines without compromising their intrinsic thermal and mechanical performance. Researchers have investigated various curing agents, including latent catalysts, acidic initiators, and co-monomers, which effectively reduce the activation energy required for ring-opening polymerization while preserving the structural integrity of the cured network [121].

Photo-initiated curing approaches have attracted increasing attention, as they enable benzoxazine polymerization under ultraviolet (UV) irradiation, significantly lowering energy consumption and processing time compared with conventional thermal curing [122,123]. In parallel, dual-curing systems—where an initial low-temperature curing step is followed by a secondary thermal or photo-curing stage—have been developed to produce highly cross-linked networks with enhanced thermal stability and mechanical robustness [124,125]. These curing innovations directly address scalability and processing challenges, thereby improving the commercial feasibility of benzoxazine-based materials, particularly for large-area coatings, flexible substrates, and textile applications.

### Challenges and prospective developments for benzoxazine-based technologies

Despite the promising performance of benzoxazines in shielding and other high-performance applications, several challenges continue to hinder their widespread industrial adoption. The relatively high cost of raw materials, combined with energy-intensive processing requirements, renders large-scale production of benzoxazine composites less economically competitive than conventional thermosetting polymers such as epoxies and phenolics [126,127]. Furthermore, the fabrication of advanced benzoxazine nanocomposites incorporating fillers such as graphene or MXenes requires additional optimization to ensure reproducibility, uniform dispersion, and cost-effectiveness [128–130].

Beyond economic constraints, achieving adequate mechanical flexibility while maintaining high thermal stability and shielding effectiveness remains a critical technical challenge. This limitation is particularly relevant for emerging applications such as wearable electronics and flexible circuit boards. Current research efforts are directed toward the incorporation of elastomers, flexible nanofillers, or toughening agents to mitigate the inherent brittleness of cured benzoxazine networks. Future work should prioritize the integration of advanced curing strategies, multifunctional additives, and sustainable material design to balance performance, flexibility, and manufacturability.

### Diverse applications of benzoxazines beyond protective functions

In addition to their well-established role in electromagnetic interference (EMI) shielding, benzoxazines exhibit characteristics that render them suitable for a broad range of advanced engineering applications. Their intrinsic flame retardancy and low moisture absorption make benzoxazine resins attractive candidates for aerospace and automotive components, where weight reduction, safety, and long-term reliability are essential requirements. Moreover, benzoxazine-based composites are increasingly utilized in thermal management systems, where efficient heat dissipation is necessary to prevent overheating and prolong the operational lifespan of electronic devices.

The excellent chemical resistance of benzoxazines further enables their use in marine and chemical environments, where protective coatings must withstand aggressive conditions [131,132]. In construction and civil engineering, benzoxazine nanocomposites reinforced with nanoclays or carbon-based fillers demonstrate enhanced mechanical strength and thermal resistance, supporting their application as structural reinforcement materials. This versatility underscores the adaptability of benzoxazine chemistry and highlights its potential for continued expansion into diverse industrial sectors.

### Health and safety considerations for the utilization of benzoxazines

As benzoxazine-based materials gain broader industrial relevance, health and safety considerations associated with their synthesis and processing must be carefully addressed. The production of benzoxazine resins often involves hazardous intermediates such as formaldehyde and aromatic amines, which may pose occupational risks if improperly handled [133–135]. Implementation of adequate ventilation systems, personal protective equipment, and controlled processing environments is therefore essential to minimize worker exposure [136–138].

Additionally, the elevated temperatures required during conventional curing may lead to the release of volatile by-products, emphasizing the need for effective fume extraction and thermal management systems in manufacturing facilities [139–141]. Although cured benzoxazine materials are generally considered stable and safe for end users, their long-term environmental impact—particularly with respect to waste management and end-of-life disposal—remains a concern. Future research should therefore emphasize safer synthesis routes, reduced reliance on toxic precursors, and the development of recycling or degradation strategies to mitigate potential health and environmental risks [142–145].

## Conclusion

Benzoxazine-based composites exhibit significant potential as advanced shielding materials, offering distinct advantages in terms of thermal stability, mechanical strength, and electromagnetic interference (EMI) shielding effectiveness. The incorporation of functional nanofillers, including carbon nanotubes, graphene, and metallic nanoparticles, has been shown to substantially enhance electrical conductivity and shielding performance, enabling compliance with stringent requirements in sectors such as electronics, aerospace, and protective textiles. The inherent resistance of benzoxazine composites to environmental stresses, including temperature fluctuations, humidity, and chemical exposure, further highlights their reliability for long-term operation, positioning them as attractive candidates for high-performance and demanding applications. From a future perspective, continued research is expected to focus on improving scalability, processing efficiency, and environmental sustainability of benzoxazine-based systems. The development of bio-based benzoxazine resins, along with low-temperature and energy-efficient curing strategies, will be critical to reducing production costs and environmental impact. Moreover, emerging concepts such as hybrid architectures and self-healing nanocomposites offer promising routes to enhance flexibility, toughness, and service life, particularly for wearable electronics and multifunctional shielding materials. Overall, this review demonstrates that benzoxazine-based composites, through rational material design and continued innovation, are well positioned to address current and future challenges in advanced shielding technologies, contributing meaningfully to both scientific advancement and industrial performance benchmarks. From the authors' perspective, future research on benzoxazine-based shielding materials should prioritize scalable low-temperature curing strategies, sustainable bio-based formulations, and the development of flexible multifunctional composites for wearable and textile applications.

## Data Availability

No new data was introduced in this work. All other data was inserted in the text.
